# Association of serum lipids with inflammatory bowel disease: a systematic review and meta-analysis

**DOI:** 10.3389/fmed.2023.1198988

**Published:** 2023-08-24

**Authors:** Hongxin Chen, Weiyang Li, Jingyi Hu, Feng Xu, Yizhou Lu, Lei Zhu, Hong Shen

**Affiliations:** ^1^Department of Gastroenterology, Affiliated Hospital of Nanjing University of Chinese Medicine, Nanjing, China; ^2^The First School of Clinical Medicine, Nanjing University of Chinese Medicine, Nanjing, China

**Keywords:** inflammatory bowel disease, Cronh’s disease, ulcerative disease, serum lipids, meta-analysis

## Abstract

**Background:**

Serum lipid levels seem to be abnormal in Inflammatory bowel disease (IBD). However, the specific manifestation of abnormal serum lipid levels in IBD are heterogeneous among studies and have not been sufficiently determined yet.

**Methods:**

PubMed, EMBASE, and Cochrane Library databases were searched. Serum lipid levels were compared between IBD patients and Health individuals, Crohn’s (CD) and ulcerative colitis (UC), active and inactive, mild and non-mild patients, respectively. Meta-analyses were performed by using a random-effect model. Weight mean difference (WMD) with 95% confidence intervals (CIs) were calculated.

**Results:**

Overall, 53 studies were included. Compared with healthy controls, IBD patients had significantly lower TC (WMD = −0.506, 95%CI = −0.674 to −0.338, *p* < 0.001), HDL-c (WMD = −0.122, 95%CI = −0.205 to −0.039, *p* = 0.004), and LDL-c (WMD = −0.371, 95%CI = −0.547 to −0.194, *p* < 0.001) levels. CD groups had a significantly lower TC (WMD = −0.349, 95%CI = −0.528 to −0.170, *p* < 0.0001) level as compared to UC groups. Active IBD and non-mild UC groups had significantly lower TC (WMD = −0.454, 95%CI = −0.722 to −0.187, *p* = 0.001) (WMD =0.462, 95%CI = 0.176 to 0.748, *p* = 0.002) and LDL-c (WMD = −0.225, 95%CI = −0.445 to −0.005, *p* = 0.045) (WMD =0.346, 95%CI = 0.084–0.609, *p* = 0.010) levels as compared to inactive IBD and mild UC groups, respectively.

**Conclusion:**

The overall level of serum lipids in IBD patients is lower than that of healthy individuals and is negatively associated with disease severity.

**Systematic review registration:**

https://www.crd.york.ac.uk/prospero/, identifier: CRD42022383885.

## 1. Introduction

Inflammatory bowel disease (IBD), including Crohn’s disease (CD) and ulcerative colitis (UC), is a chronic disease that mainly causes inflammation of the gastrointestinal tract (1). Its global prevalence is more than 0.3%, and the incidence and prevalence are still increasing worldwide (2). The specific pathogenesis of IBD remains unclear, but it seems to be a disruption of intestinal homeostasis caused by complex interactions among susceptible genes, inappropriate diet and immune response, and environmental risk factors (3, 4).

In this study, serum lipids mainly include total cholesterol (TC), high density lipoprotein cholesterol (HDL-c), low density lipoprotein cholesterol (LDL-c), and triglyceride (TG) (5). Normally, the main function of serum lipids is to maintain the body’s energy metabolism, synthesize cell membranes, steroid hormones, and bile acids. When autoimmunity and chronic inflammation occur in the human body, lipoprotein metabolism will be impaired and altered, causing various changes in serum lipid profiles (6–8). For example, systemic lupus erythematosus, a chronic inflammatory disease, is characterized by the presence of proinflammatory cytokines and anti-lipoprotein lipase antibodies, leading to a characteristic “lupus pattern” of lipoproteins, which mainly manifested by elevated TG and decreased HDL-c levels (6, 9, 10). Furthermore, abnormal serum lipid levels can stimulate the release of inflammatory mediators, aggravate inflammation, and promote disease progression (11). Additionally, when inflammation involves the intestine, it may affect the body’s absorption and metabolism of lipids, resulting in malabsorption of nutrients and fats, which in turn affect serum lipids metabolism (12). IBD, as a chronic, autoimmune, and inflammatory disease, may also have its own unique characteristics of serum lipid changes. However, in the current study, there is heterogeneity in the results of serum lipid levels in IBD patients. Some studies found that patients with IBD had low serum lipid levels than those without (12–14). By contrast, other studies found that patients with IBD had a high TG or HDL-c level than those without (15–17). More notably, no one meta-analysis has yet explored their association. Therefore, we have comprehensively collected relevant data and conducted a meta-analysis to analyze the correlation between serum lipids and IBD, aiming to explore the unique serum lipid profile of IBD.

## 2. Methods

The meta-analysis was performed based on the Preferred Reporting Items for Systematic Reviews and Meta-Analyses (PRISMA) statement. The PRISMA checklist is shown in Supplementary material 1.

### 2.1. Registration

The meta-analysis was registered in PROSPERO with a registration number of CRD42022383885.

### 2.2. Literature search

PubMed Medline, Embase, and Cochrane Library were searched. Searched items are listed in Supplementary material 2. The last search was performed on March 7, 2023. There was no language limitation.

### 2.3. Selection criteria

All studies regarding the data of serum lipids in IBD, CD, and UC were included. Exclusion criteria were as follows: (1) duplicated studies; (2) reviews and meta-analyses; (3) case reports; (4) guidelines, consensus, or reports; (5) experimental or animal studies; (6) irrelevant papers; (7) comments, letters, or notes; (8) participants with dyslipidemia; (9) combine with comorbidities; (10) overlapping participants among studies; and (11) absence of relevant data.

### 2.4. Outcomes of interest

The primary outcome should be explored differences in the manifestation of serum lipid levels between IBD and healthy controls, which included IBD versus healthy controls; UC versus healthy controls; and CD versus healthy controls, respectively. The secondary outcomes should be explored differences in the manifestation of serum lipid levels by disease type and severity, respectively.

### 2.5. Data extraction

The following data were extracted from the included studies: first author, publication year, region, type of publication, study design, enrollment period, type and severity of IBD, number and age of participants in case and control groups, and the levels of TC, HDL-c, LDL-c, and TG at baseline.

### 2.6. Study quality assessment

The quality of case–control and cohort studies was assessed by the Newcastle-Ottawa Scale (NOS), which includes 3 parts (i.e., Selection, Comparability, and exposure) and 8 questions with the highest score of 9 stars. A score of 0–3, 4–6, and 7–9 represents low, moderate, and high quality, respectively. The quality of cross-sectional studies was assessed with 11 items formulated by the Agency for Healthcare Research and Quality (AHRQ), which are answered with “yes,” “no,” or “unclear.” The maximum AHRQ score is 11. A score of 0–3, 4–7, and 8–11 represents low, moderate, and high quality, respectively.

### 2.7. Disease assessment

According to included studies, CD activity was assessed mainly according to Crohn’s Disease Activity Index scores (16, 18–22) or Harvey-Bradshaw scores (23, 24), and UC activity and severity were assessed mainly according to the modified Mayo score (25) or Truelove-Witts Severity Index (18, 20, 23, 26) or the simple clinical colitis activity index (16).

### 2.8. Statistical analysis

The meta-analysis was performed by the Review Manager 5.2 (Cochrane collaboration, the Nordic Cochrane Centre, Copenhagen, Denmark) and STATA 12.0 (Stata Corp, College Station, Texas, United States). A random-effect model was employed. *p-*value < 0.05 was considered statistically significant. Continuous variables will be expressed as weight mean difference (WMD) with 95% confidence intervals (CIs). If continuous variables will be expressed as median with range or interquartile, we will use the Box-Cox method to convert them to mean with standard deviation (27). The Cochrane Q test and I^2^ statistics were employed to assess the heterogeneity. *I*^2^ > 50% and/or *p* < 0.1 were considered to have statistically significant heterogeneity. Publication bias was performed with Egger test. *p* < 0.1 was considered as a statistically significant publication bias. Subgroup analyses were conducted according to IBD types (UC or CD). The meta-regression analyses and sensitivity analyses were used to explore the sources of heterogeneity. Covariates used for meta-regression analyses included study design (case–control vs. cross-sectional vs. cohort), publication year (before 2010 vs. after 2010), region (Asia vs. Europe vs. America vs. Oceania), sample size (≤100 vs. >100), and whether age and gender were matched between patients with and without IBD (matched vs. unmatched). Leave-one-out sensitivity analyses were assessed by sequentially omitting a single study in turn.

## 3. Results

### 3.1. Study selection

**Figure 1:**
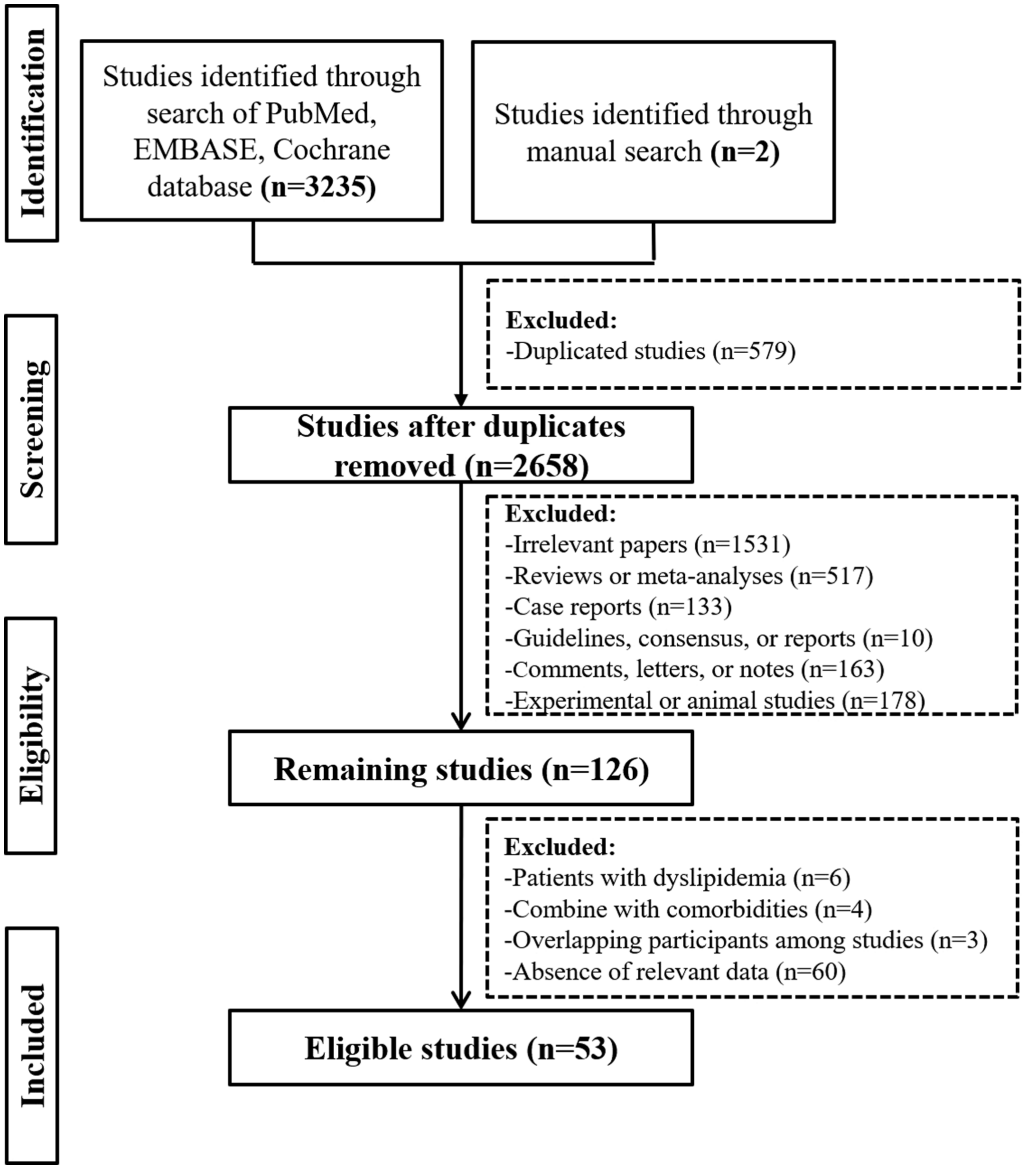
A flowchart of study inclusion.

Overall, the initial search identified 3,235 studies from the PubMed, EMBASE, and Cochrane Library databases, and 2 study from hand-searching. Finally, 53 studies were included (Figure 1).

### 3.2. Study characteristics

Characteristics of the included studies were shown in Table 1. Among them, 24 studies were case–control studies, 4 studies were cohort studies, and 25 were cross-sectional studies. All of them were published between 1979 and 2022. In addition, 17 studies were performed in Asia (13, 14, 19, 20, 25, 26, 33–36, 43, 47, 51–53, 59, 60), 30 in Europe (15–18, 21, 24, 28–32, 37, 39–42, 44, 46, 48, 50, 55–58, 61–66), 5 in America (22, 23, 38, 45, 54), and 1 in Oceania (49).

**Table 1:** Characteristics of included studies regarding serum lipid levels in inflammatory bowel disease.

Reference	Region	Study design	Type of publication	Enrollment period	Type of patients	Number of patients	Age, year (Mean ± SD)	TC, mmol/L (Mean ± SD)	HDL-c, mmol/L (Mean ± SD)	LDL-c, mmol/L (Mean ± SD)	TG, mmol/L (Mean ± SD)
Sleutjes Am et al. (28)	Netherlands	Cross-sectional	Abstract	NA	IBD vs. HC	217/829	NA	4.3 ± 16.2 vs. 5.4 ± 31.7	1.2 ± 7.4 vs. 1.2 ± 11.5	2.6 ± 16.2 vs. 3.8 ± 28.8	2.7 ± 11.8 vs. 3.63 ± 23.0
Lu et al. (14)	China	Cross-sectional	Full text	2014.03–2020.08	CD vs. HC	862/576	33 ± 13 vs. 34 ± 11	3.71 ± 0.86 vs. 5.04 ± 0.94	0.94 ± 0.27 vs. 1.31 ± 0.33	1.85 ± 0.59 vs. 2.92 ± 0.81	1.10 ± 0.40 vs. 1.64 ± 1.02
Hernández-Camba et al. (17)	Spain	Cross-sectional	Full text	NA	IBD vs. HC	197/208	50 ± 15 vs. 49 ± 10	5.25 ± 1.27 vs. 5.12 ± 1.16	1.47 ± 0.47 vs. 1.32 ± 0.36	3.00 ± 1.03 vs. 3.05 ± 0.96	1.31 ± 0.45 vs. 1.63 ± 0.79
Wang et al. (13)	China	Cross-sectional	Full text	2014.1–2018.11	IBD vs. HC	539/165	40 ± 16 vs. 50 ± 11	3.87 ± 1.01 vs. 4.90 ± 0.90	1.03 ± 0.99 vs. 1.18 ± 0.31	2.41 ± 0.70 vs. 3.14 ± 0.65	1.09 ± 0.42 vs. 1.62 ± 0.91
					CD vs. UC	307/232	34 ± 13 vs. 48 ± 16	3.70 ± 0.90 vs. 4.10 ± 1.10	1.00 ± 0.26 vs. 1.08 ± 0.32	2.29 ± 0.64 vs. 2.58 ± 0.74	1.04 ± 0.41 vs. 1.15 ± 0.43
Li et al. (25)	China	Cross-sectional	Full text	2018.06–2019.04	Mild active UC vs. Non-mild active UC	22/24	43 ± 3 vs. 46 ± 3	3.97 ± 0.82 vs. 3.63 ± 0.76	NA	2.59 ± 0.83 vs. 2.33 ± 0.73	NA
Carrillo-Palau et al. (29)	Spain	Cross-sectional	Full text	NA	IBD vs. HC	151/174	48 ± 10 vs. 50 ± 16	5.07 ± 1.14 vs. 5.28 ± 1.09	1.42 ± 0.41 vs. 1.40 ± 0.39	2.92 ± 0.96 vs. 3.15 ± 0.88	1.59 ± 0.90 vs. 1.56 ± 0.82
Vrdoljak et al. (30)	Croatia	Cross-sectional	Full text	2017.12–2019.04	CD vs. UC	50/44	38 ± 13 vs. 44 ± 14	4.3 ± 1.26 vs. 5.4 ± 1.43	1.28 ± 0.4 vs. 1.5 ± 0.48	2.35 ± 0.89 vs. 3.38 ± 1.27	1.48 ± 1.36 vs. 1.12 ± 0.66
Brnić et al. (31)	Croatia	Cross-sectional	Full text	2017.12.01–2018.06.01	IBD vs. HC	55/50	39 ± 14 vs. 37 ± 13	5.02 ± 1.52 vs. 5.25 ± 1.19	1.37 ± 0.43 vs. 1.41 ± 0.32	2.98 ± 1.20 vs. 3.25 ± 1.10	1.33 ± 1.26 vs. 1.24 ± 0.60
Dragasevic et al. (32)	Serbia	Cross-sectional	Full text	NA	IBD vs. HC	104/45	40 ± 16 vs. 43 ± 18	4.37 ± 1.17 vs. 4.97 ± 1.00	1.06 ± 0.74 vs. 1.70 ± 0.67	2.16 ± 0.74 vs. 2.72 ± 0.87	1.43 ± 1.06 vs. 1.10 ± 0.59
					CD vs. UC	50/54	38 ± 13 vs. 44 ± 14	4.34 ± 1.09 vs. 4.39 ± 1.25	1.02 ± 0.68 vs. 1.11 ± 0.79	1.97 ± 0.74 vs. 2.33 ± 0.71	1.44 ± 0.95 vs. 1.43 ± 1.16
Sahin et al. (33)	Turkey	Cross-sectional	Full text	2016.01–2016.09	UC vs. HC	66/24	40 ± 12 vs. 46 ± 18	NA	1.26 ± 0.39 vs. 1.28 ± 0.35	2.55 ± 1.04 vs. 2.95 ± 1.31	NA
Qiao et al. (34)	China	Cross-sectional	Full text	2015.01–2015.12	CD vs. UC	129/69	35 ± 11 vs. 44 ± 15	3.36 ± 0.79 vs. 3.82 ± 1.26	NA	NA	1.07 ± 0.54 vs. 1.10 ± 0.86
Mańkowska-Wierzbicka et al. (18)	Poland	Cohort	Full text	NA	CD vs. UC	34/31	NA	3.35 ± 1.02 vs. 3.47 ± 1.27	0.96 ± 0.45 vs. 1.04 ± 0.37	1.70 ± 0.77 vs. 1.04 ± 0.37	1.18 ± 0.45 vs. 1.08 ± 0.39
					Active CD vs. Active UC	22/19		3.34 ± 1.03 vs. 3.39 ± 1.33	0.96 ± 0.42 vs. 1.05 ± 0.40	1.71 ± 0.80 vs. 1.88 ± 0.87	1.18 ± 0.44 vs. 1.21 ± 0.41
Iwakawa et al. (35)	Japan	Cross-sectional	Full text	2010.08–2010.10	Active UC vs. Inactive UC	6/17	45 ± 16 vs. 42 ± 15	4.86 ± 1.27 vs. 5.04 ± 0.65	NA	NA	NA
Kang et al. (36)	Korea	Cohort	Full text	2010.01–2014.11	IBD vs. HC	8070/40350	45 ± 13 vs. 45 ± 13	4.72 ± 0.92 vs. 4.96 ± 0.92	NA	NA	NA
Aarestrup et al. (37)	Denmark	Cohort	Full text	NA	IBD vs. HC	1203/107586	57 ± 3 vs. 58 ± 3	5.71 ± 1.14 vs. 5.81 ± 1.14	1.60 ± 0.59 vs. 1.57 ± 0.50	3.20 ± 0.98 vs. 3.30 ± 0.89	1.50 ± 0.80 vs. 1.68 ± 1.02
Trejo-Vazquez et al. (38)	Mexico	Case-control	Full text	2016.07–2016.10	IBD vs. HC	34/19	55 ± 15 vs. 53 ± 10	4.68 ± 0.74 vs. 4.95 ± 1.07	1.17 ± 0.32 vs. 1.18 ± 0.32	2.86 ± 0.71 vs. 3.19 ± 0.85	1.49 ± 0.58 vs. 1.36 ± 0.43
Szczeklik et al. (39)	Poland	Case-control	Full text	NA	CD vs. HC	58/25	36 ± 13 vs. 34 ± 10	4.35 ± 0.66 vs. 4.73 ± 0.39	0.87 ± 0.25 vs. 1.14 ± 0.14	2.38 ± 0.49 vs. 2.95 ± 0.38	1.85 ± 0.31 vs. 2.76 ± 0.27
Schulte et al. (15)	Germany	Case-control	Full text	NA	IBD vs. HC	35/35	39 ± 25 vs. 39 ± 24	4.75 ± 0.81 vs. 5.13 ± 1.05	1.38 ± 0.49 vs. 1.49 ± 0.35	2.69 ± 0.61 vs. 3.02 ± 0.92	1.53 ± 0.62 vs. 1.11 ± 0.63
Grzybowska-Chlebowczyk et al. (40)	Poland	Cross-sectional	Full text	Two years	CD vs. UC	35/36	16 ± 2 vs. 14 ± 4	3.30 ± 0.60 vs. 3.60 ± 0.84	NA	1.74 ± 0.81 vs. 1.84 ± 0.70	1.74 ± 0.82 vs. 0.97 ± 0.43
Trzeciak-Jędrzejczyk et al. (41)	Poland	Case-control	Full text	NA	IBD vs. HC	40/11	NA	3.16 ± 0.53 vs. 3.82 ± 0.79	1.10 ± 0.46 vs. 1.55 ± 0.57	1.70 ± 0.44 vs. 1.83 ± 0.46	0.94 ± 0.41 vs. 1.12 ± 0.29
					CD vs. UC	25/15	NA	3.22 ± 0.51 vs. 2.90 ± 0.52	1.17 ± 0.46 vs. 0.99 ± 0.46	1.82 ± 0.35 vs. 1.50 ± 0.51	0.85 ± 0.22 vs. 1.10 ± 0.59
Cappello et al. (42)	Italy	Case-control	Full text	2012.09–2013.12	IBD vs. HC	68/38	44 ± 13 vs. 41 ± 11	4.14 ± 0.85 vs. 4.57 ± 0.92	1.35 ± 0.40 vs. 1.47 ± 0.47	2.33 ± 0.70 vs. 2.67 ± 0.87	1.06 ± 0.45 vs. 0.92 ± 0.51
Üstün et al. (43)	Turkey	Case-control	Full text	2007.03–2009.10	IBD vs. HC	96/65	44 ± 13 vs. 41 ± 11	4.79 ± 1.26 vs. 4.90 ± 1.00	NA	2.72 ± 0.93 vs. 2.84 ± 0.71	1.51 ± 0.81 vs. 1.49 ± 0.78
Qin et al. (19)	China	Case-control	Full text	2013.11–2015.07	CD vs. HC	100/100	33 ± 13 vs. 35 ± 10	3.56 ± 0.91 vs. 4.65 ± 0.72	0.96 ± 0.23 vs. 1.49 ± 0.32	1.99 ± 0.66 vs. 2.80 ± 0.57	1.11 ± 0.44 vs. 1.32 ± 0.53
					Active CD vs. Inactive CD	62/38	33 ± 12 vs. 33 ± 14	3.40 ± 0.78 vs. 3.83 ± 1.05	0.93 ± 0.22 vs. 1.01 ± 0.23	1.87 ± 0.54 vs. 2.19 ± 0.78	1.06 ± 0.35 vs. 1.21 ± 0.55
Pac-Kożuchowska et al. (44)	Poland	Case-control	Full text	NA	IBD vs. HC	30/20	13 ± 3 vs. 13 ± 4	3.40 ± 0.66 vs. 3.20 ± 0.73	1.17 ± 0.35 vs. 1.06 ± 0.24	1.99 ± 0.53 vs. 1.96 ± 0.62	0.97 ± 0.37 vs. 0.81 ± 0.30
Koutroumpakis et al. (45)	USA	Cohort	Full text	2009.1–2014.10	CD vs. UC	380/321	33 ± 18 vs. 35 ± 18	4.40 ± 0.90 vs. 4.70 ± 0.98	1.31 ± 0.43 vs. 1.36 ± 0.41	2.34 ± 0.88 vs. 2.76 ± 0.82	1.64 ± 0.99 vs. 1.38 ± 0.88
De Fatima and Bodanese (23)	Brazil	Cross-sectional	Full text	2014.10–2015.11	Active CD vs. Active UC	64/58	42 ± 13 vs. 42 ± 12	4.39 ± 1.03 vs. 4.77 ± 1.10	1.38 ± 0.35 vs. 1.45 ± 0.42	2.36 ± 0.86 vs. 2.84 ± 0.93	1.46 ± 0.99 vs. 1.26 ± 0.59
Aguilar-Tablada et al. (46)	Spain	Case-control	Full text	NA	CD vs. UC	53/53	NA	5.02 ± 1.21 vs. 4.23 ± 0.99	NA	NA	NA
Wada et al. (47)	Japan	Cross-sectional	Full text	2009–2010	CD vs. UC	156/232	36 ± 8 vs. 36 ± 8	4.11 ± 0.99 vs. 4.88 ± 0.88	NA	NA	NA
Aytac et al. (20)	Turkey	Case-control	Full text	NA	Inactive IBD vs. HC	55/25	42 ± 11 vs. 42 ± 7	4.32 ± 0.80 vs. 4.37 ± 0.46	1.19 ± 0.25 vs. 1.18 ± 0.24	2.68 ± 0.70 vs. 2.50 ± 0.46	0.96 ± 0.36 vs. 1.53 ± 0.48
					Inactive CD vs. Inactive UC	25/30	45 ± 12 vs. 39 ± 10	4.00 ± 0.87 vs. 4.58 ± 0.64	1.14 ± 0.31 vs. 1.23 ± 0.17	2.59 ± 0.77 vs. 2.76 ± 0.64	1.01 ± 0.28 vs. 0.91 ± 0.41
Theocharidou et al. (48)	Greece	Case-control	Full text	NA	IBD vs. HC	44/44	36 ± 10 vs. 37 ± 11	4.49 ± 1.21 vs. 5.20 ± 0.94	1.26 ± 0.39 vs. 1.31 ± 0.35	2.75 ± 0.94 vs. 3.35 ± 0.82	0.99 ± 0.37 vs. 1.11 ± 0.56
Fan et al. (49)	Australia	Case-control	Full text	NA	IBD vs. HC	42/73	50 ± 10 vs. 51 ± 10	5.23 ± 1.20 vs. 5.45 ± 0.86	1.46 ± 0.45 vs. 1.50 ± 0.33	3.19 ± 1.15 vs. 3.45 ± 0.81	1.34 ± 1.06 vs. 1.11 ± 0.65
Principi et al. (50)	Italy	Case-control	Full text	2011.05–2011.10	IBD vs. HC	49/40	41 ± 16 vs. 45 ± 15	4.29 ± 0.52 vs. 4.42 ± 0.85	1.21 ± 0.18 vs. 1.26 ± 0.31	2.48 ± 0.49 vs. 2.59 ± 0.85	1.31 ± 0.15 vs. 1.26 ± 0.65
					CD vs. UC	26/23	36 ± 17 vs. 45 ± 14	4.27 ± 0.57 vs. 4.34 ± 0.47	1.21 ± 0.21 vs. 1.24 ± 0.18	2.46 ± 0.49 vs. 2.48 ± 0.49	1.30 ± 0.12 vs. 1.31 ± 0.16
Liu et al. (26)	China	Cross-sectional	Full text	2006.01–2012.11	Active UC vs. HC	97/100	56 ± 15 vs. 59 ± 13	4.20 ± 0.95 vs. 4.60 ± 1.10	1.13 ± 0.33 vs. 1.29 ± 0.33	2.61 ± 0.82 vs. 2.64 ± 0.78	1.44 ± 1.00 vs. 1.43 ± 1.01
					Mild active UC vs. Non-mild active UC	41/56	NA	4.51 ± 0.88 vs. 3.97 ± 0.95	1.23 ± 0.29 vs. 1.05 ± 0.33	2.84 ± 0.80 vs. 2.45 ± 0.80	1.43 ± 1.04 vs. 1.44 ± 0.98
Akdoğan et al. (51)	Turkey	Cross-sectional	Full text	NA	UC vs. HC	37/30	48 ± 15 vs. 45 ± 8	5.07 ± 0.98 vs. 5.12 ± 1.19	1.16 ± 0.28 vs. 1.16 ± 0.23	3.49 ± 1.01 vs. 3.26 ± 0.93	1.52 ± 0.75 vs. 1.52 ± 0.94
Yorulmaz et al. (52)	Turkey	Cross-sectional	Full text	NA	CD vs. UC	62/115	37 ± 14 vs. 44 ± 14	NA	1.37 ± 0.40 vs. 1.40 ± 0.39	NA	1.37 ± 0.72 vs. 1.37 ± 0.65
Kuwabara et al. (53)	Japan	Cross-sectional	Full text	NA	CD vs. UC	33/31	36 ± 7 vs. 42 ± 17	3.28 ± 0.65 vs. 4.58 ± 1.04	NA	NA	NA
Sappati Biyyani et al. (54)	USA	Cross-sectional	Full text	2000.01–2007.12	CD vs. UC	190/204	49 ± 13 vs. 49 ± 14	4.52 ± 1.04 vs. 4.79 ± 1.04	1.25 ± 0.35 vs. 1.27 ± 0.36	2.93 ± 0.89 vs. 3.06 ± 0.84	1.31 ± 0.77 vs. 1.40 ± 1.06
Mijac et al. (55)	Serbia	Cross-sectional	Full text	NA	IBD vs. HC	76/30	41 ± 15 vs. 45 ± 18	3.80 ± 1.17 vs. 4.85 ± 0.82	NA	NA	1.54 ± 1.71 vs. 2.11 ± 0.87
					CD vs. UC	23/53	39 ± 15 vs. 42 ± 15	3.54 ± 0.96 vs. 3.90 ± 1.24	NA	NA	1.92 ± 2.82 vs. 1.40 ± 1.05
Romanato et al. (56)	Italy	Cross-sectional	Full text	2004.12–2006.03	IBD vs. HC	94/94	NA	4.20 ± 1.09 vs. 5.35 ± 0.63	1.30 ± 0.44 vs. 1.40 ± 0.25	2.37 ± 0.98 vs. 3.34 ± 0.57	1.18 ± 0.53 vs. 1.33 ± 0.33
					CD vs. UC	60/34	45 ± 23 vs. 50 ± 13	4.16 ± 1.15 vs. 4.27 ± 1.00	1.32 ± 0.45 vs. 1.26 ± 0.48	2.35 ± 1.00 vs. 2.40 ± 0.96	1.11 ± 0.43 vs. 1.29 ± 0.66
Hrabovský et al. (21)	Czech Republic	Case-control	Full text	NA	Active CD vs. HC	24/100	NA	3.16 ± 1.16 vs. 4.90 ± 0.98	NA	NA	NA
Scarpa et al. (57)	Italy	Case-control	Full text	2004.12–2006.03	UC vs. HC	15/15	50 ± 29 vs. 50 ± 28	4.19 ± 1.29 vs. 5.56 ± 1.05	1.09 ± 0.40 vs. 1.46 ± 0.42	2.47 ± 1.15 vs. 3.51 ± 0.98	1.18 ± 0.40 vs. 1.32 ± 0.83
Van Leuven et al. (24)	Netherlands	Case-control	Full text	NA	CD vs. HC	60/122	42 ± 12 vs. 41 ± 16	4.54 ± 1.12 vs. 5.04 ± 0.99	1.53 ± 0.48 vs. 1.47 ± 0.53	2.59 ± 0.96 vs. 2.99 ± 0.81	0.92 ± 0.62 vs. 1.34 ± 1.25
					Active CD vs. Inactive CD	12/48	34 ± 9 vs. 44 ± 13	3.79 ± 0.89 vs. 4.73 ± 1.11	1.01 ± 0.30 vs. 1.66 ± 0.43	2.46 ± 0.91 vs. 2.62 ± 0.97	0.70 ± 0.88 vs. 0.98 ± 0.54
Figler et al. (58)	Hungary	Cross-sectional	Full text	NA	IBD vs. HC	51/24	40 ± 12 vs. 32 ± 9	5.16 ± 1.15 vs. 5.61 ± 0.94	1.58 ± 0.39 vs. 1.58 ± 0.39	NA	NA
					Inactive CD vs. Inactive UC	21/30	38 ± 11 vs. 41 ± 12	4.75 ± 0.95 vs. 5.48 ± 1.20	1.60 ± 0.45 vs. 1.57 ± 0.35	NA	NA
Yılmaz et al. (59)	Turkey	Case-control	Full text	NA	IBD vs. HC	33/27	34 ± 15 vs. 34 ± 11	4.55 ± 0.65 vs. 4.32 ± 0.77	1.09 ± 0.22 vs. 1.22 ± 0.21	2.61 ± 0.66 vs. 2.56 ± 0.73	1.50 ± 0.57 vs. 1.43 ± 0.95
Ripollés Piquer et al. (16)	France	Case-control	Full text	NA	IBD vs. HC	21/28	29 ± 9 vs. 31 ± 9	4.26 ± 1.21 vs. 5.25 ± 1.06	1.28 ± 0.28 vs. 1.78 ± 0.47	2.42 ± 1.04 vs. 3.10 ± 0.88	1.21 ± 0.54 vs. 0.45 ± 0.46
					Active IBD vs. Inactive IBD	15/6	28 ± 9 vs. 33 ± 11	4.19 ± 1.32 vs. 4.42 ± 0.96	1.24 ± 0.23 vs. 1.38 ± 0.38	2.40 ± 1.18 vs. 2.48 ± 0.65	1.21 ± 0.60 vs. 1.22 ± 0.40
Tajika et al. (60)	Japan	Cross-sectional	Full text	2001.12–2002.1	IBD vs. HC	44/15	40 ± 10 vs. 38 ± 10	4.20 ± 1.26 vs. 5.28 ± 0.83	NA	NA	NA
					CD vs. UC	33/11	38 ± 8 vs. 48 ± 12	3.80 ± 1.11 vs. 5.42 ± 0.87	NA	NA	NA
Koutroubakis et al. (61)	Greece	Case-control	Full text	NA	IBD vs. HC	129/66	NA	5.15 ± 1.59 vs. 6.11 ± 1.37	1.26 ± 0.40 vs. 1.23 ± 0.32	3.32 ± 1.33 vs. 4.11 ± 1.12	1.31 ± 0.70 vs. 1.42 ± 0.91
					CD vs. UC	66/63	NA	5.57 ± 1.52 vs. 4.71 ± 1.57	1.30 ± 0.40 vs. 1.22 ± 0.40	3.75 ± 1.19 vs. 2.87 ± 1.33	1.25 ± 0.54 vs. 1.38 ± 0.83
Levy et al. (22)	Canada	Case-control	Full text	NA	Active CD vs. Inactive CD	13/8	NA	3.23 ± 0.83 vs. 3.39 ± 0.62	0.93 ± 0.43 vs. 1.04 ± 0.23	1.83 ± 0.61 vs. 1.84 ± 0.59	1.04 ± 0.36 vs. 1.13 ± 0.28
Hudson et al. (62)	England	Case-control	Full text	NA	IBD vs. HC	110/85	44 ± 18 vs. 42 ± 16	NA	NA	NA	1.19 ± 0.59 vs. 1.19 ± 0.85
					CD vs. UC	75/35	44 ± 18 vs. 44 ± 18	NA	NA	NA	1.08 ± 0.48 vs. 1.41 ± 0.74
Hakala et al. (63)	Finland	Case-control	Full text	NA	CD vs. UC	29/50	31 ± 3 vs. 35 ± 3	3.84 ± 0.38 vs. 4.46 ± 0.30	1.23 ± 0.23 vs. 1.10 ± 0.10	NA	1.07 ± 0.14 vs. 1.23 ± 0.21
Regöly-Mérei et al. (64)	Hungary	Case-control	Full text	NA	UC vs. HC	11/20	NA	4.89 ± 1.39 vs. 4.06 ± 0.93	NA	NA	1.64 ± 0.80 vs. 0.93 ± 0.51
Rutgeerts et al. (65)	Belgium	Case-control	Full text	NA	CD vs. HC	56/21	29 ± 7 vs. 29 ± 11	6.00 ± 0.98 vs. 4.46 ± 1.33	NA	NA	1.08 ± 0.27 vs. 1.09 ± 0.29
Johansson et al. (66)	Sweden	Case-control	Full text	NA	CD vs. HC	37/117	NA	4.33 ± 0.96 vs. 6.55 ± 1.31	NA	NA	1.43 ± 0.58 vs. 1.41 ± 0.55

TC, total cholesterol; HDL-c, high-density lipoprotein cholesterol; LDL-c, low-density lipoprotein cholesterol; TG, triglycerides; IBD, inflammatory bowel disease; CD, Crohn’s disease; UC, ulcerative colitis; HC, healthy controls; NA, not available.

### 3.3. Study quality

Among the case–control and cohort studies, 8 and 20 were of moderate and high quality, respectively (Supplementary Table S1). Among the cross-sectional studies, 22 and 3 were of moderate and high quality, respectively (Supplementary Table S2).

### 3.4. Meta-analysis of serum lipid levels between IBD versus healthy controls

#### 3.4.1. Total cholesterol level

**Figure 2:**
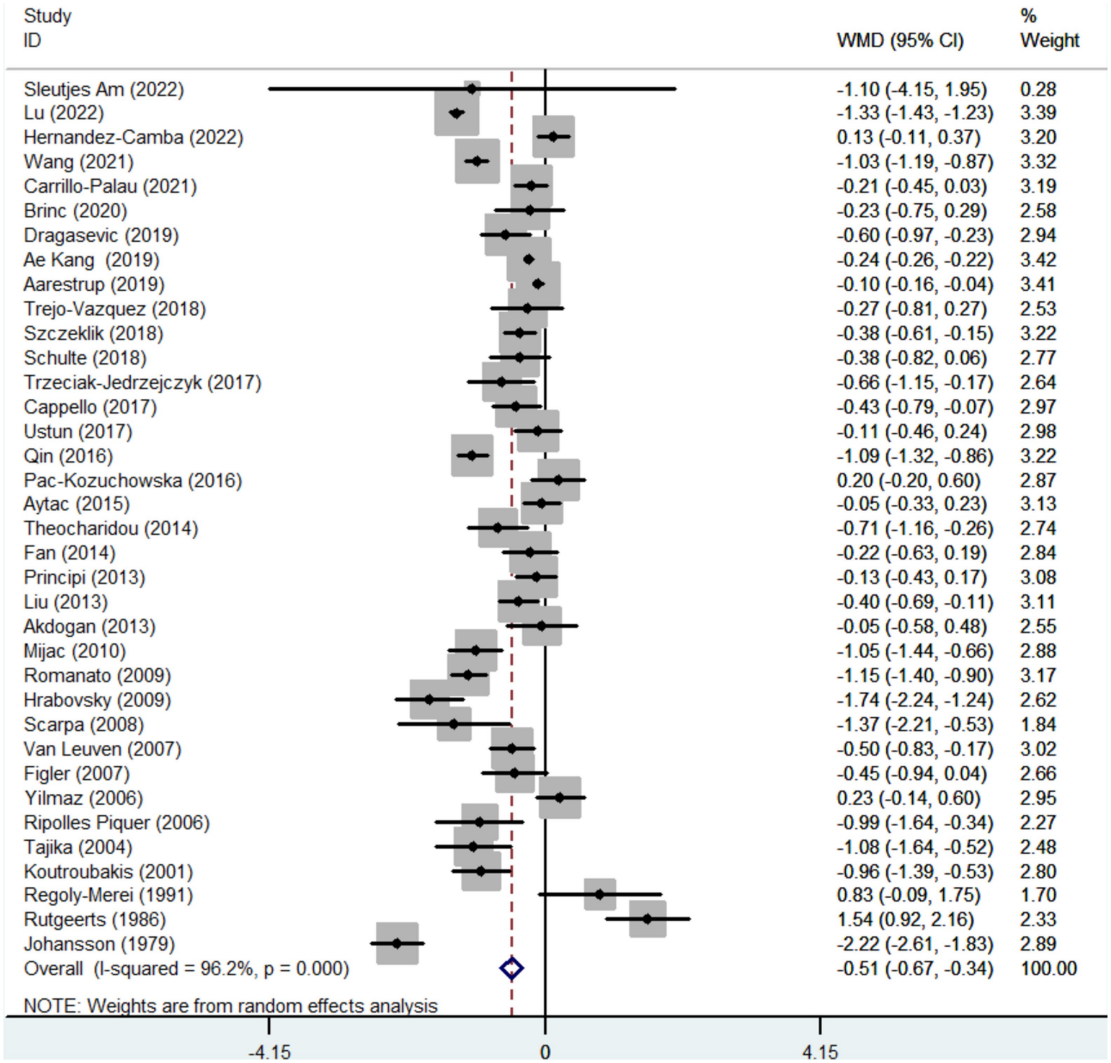
Forest plots showing the TC level between IBD and healthy controls.

Thirty-six studies reported the data regarding the TC level. Meta-analysis demonstrated that IBD groups had a significantly lower level of TC than healthy control groups (WMD = −0.506, 95%CI = −0.674 to −0.338, *p* < 0.001) (Figure 2). The heterogeneity was significant (*I*^2^ = 96.2%, *p* < 0.001). Sensitivity analysis did not find the source of heterogeneity (Supplementary Figure S1A). Meta-regression analyses found that the source of heterogeneity might be the sample size (Supplementary Table S3).

In the subgroup analyses of IBD types, 17 and 14 studies reported the data regarding the TC level in CD groups and healthy control groups, and UC groups and healthy control groups, respectively. Compared with the control group, the level of TC was also significantly lower in both CD and UC group (WMD = −0.844, 95%CI = −1.121 to −0.567, *p* < 0.001) (WMD = −0.490, 95%CI = −0.775 to −0.205, *p* = 0.001) (Supplementary Figures S2A, S4A). The heterogeneity was significant (*I*^2^ = 93.3%, *p* < 0.001) (*I*^2^ = 85.0%, *p* < 0.001). Sensitivity analysis did not find the source of heterogeneity (Supplementary Figures S3A, S5A). In UC groups versus healthy control groups, but not CD groups versus healthy control groups, meta-regression analyses found that the source of heterogeneity might be the sample size (Supplementary Table S3).

#### 3.4.2. High density lipoprotein cholesterol level

**Figure 3:**
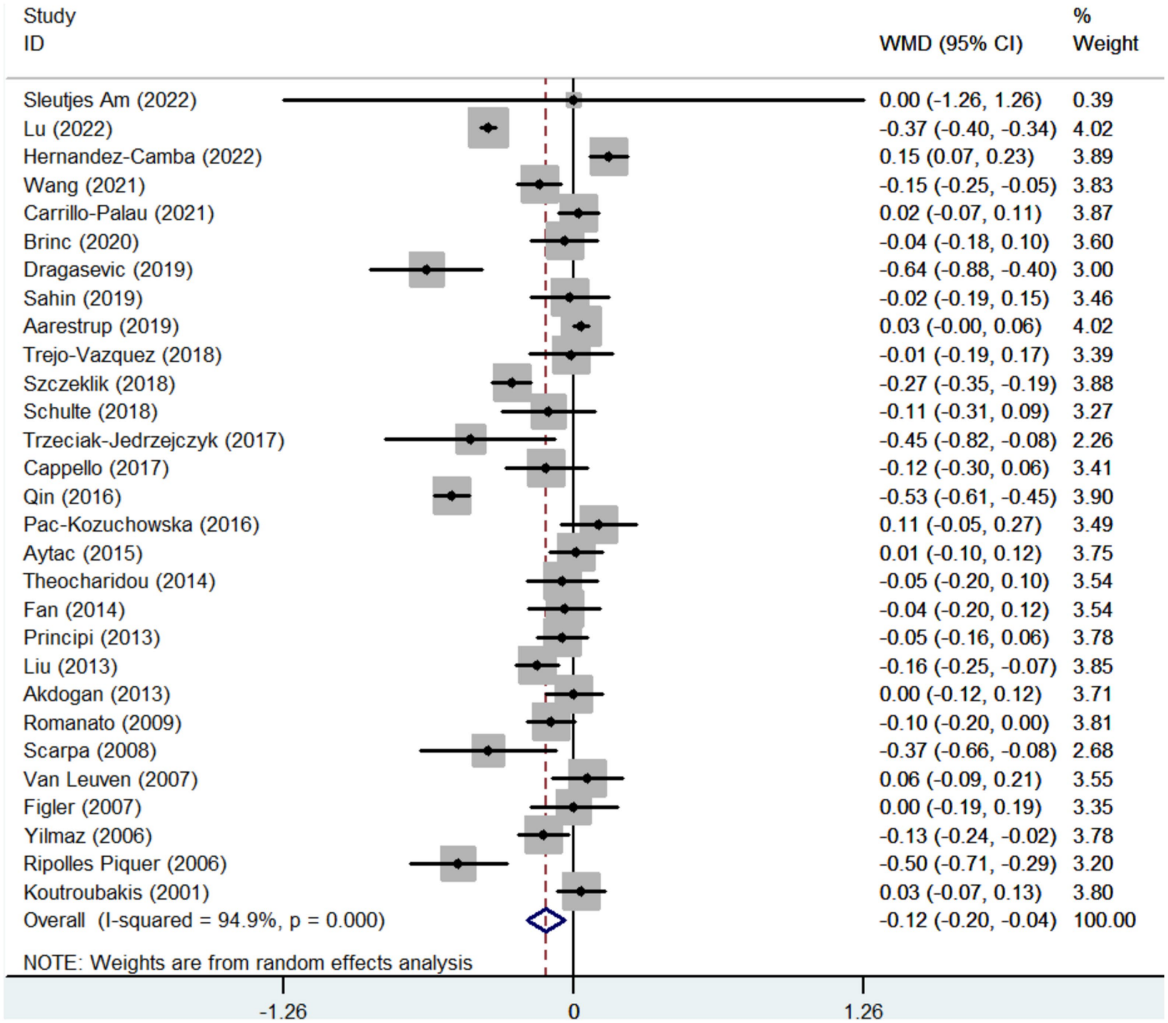
Forest plots showing the HDL-c level between IBD and healthy controls.

Twenty-nine studies reported the data regarding the HDL-c level. Meta-analysis demonstrated that IBD groups had a significantly lower level of HDL-c than healthy control groups (WMD = −0.122, 95%CI = −0.205 to −0.039, *p* = 0.004) (Figure 3). The heterogeneity was significant (*I*^2^ = 94.9%, *p* < 0.001). Sensitivity analysis and meta-regression did not find the source of heterogeneity (Supplementary Figure S1B; Supplementary Table S3).

In the subgroup analyses of IBD types, 12 and 12 studies reported the data regarding the HDL-c level in CD groups and healthy control groups, and UC groups and healthy control groups, respectively. Compared with control groups, the level of HDL-c was also significantly lower in both CD and UC groups (WMD = −0.193, 95%CI = −0.305 to −0.081, *p* = 0.001) (WMD = −0.100, 95%CI = −0.172 to −0.027, *p* = 0.007) (Supplementary Figures S2B, S4B). The heterogeneity was significant (I^2^ = 93.8%, *p* < 0.001) (*I*^2^ = 68.3%, *p* < 0.0001). Sensitivity analysis and meta-regression analyses did not find the source of heterogeneity (Supplementary Figures S3B, S5B; Supplementary Table S3).

#### 3.4.3. Low density lipoprotein cholesterol level

**Figure 4:**
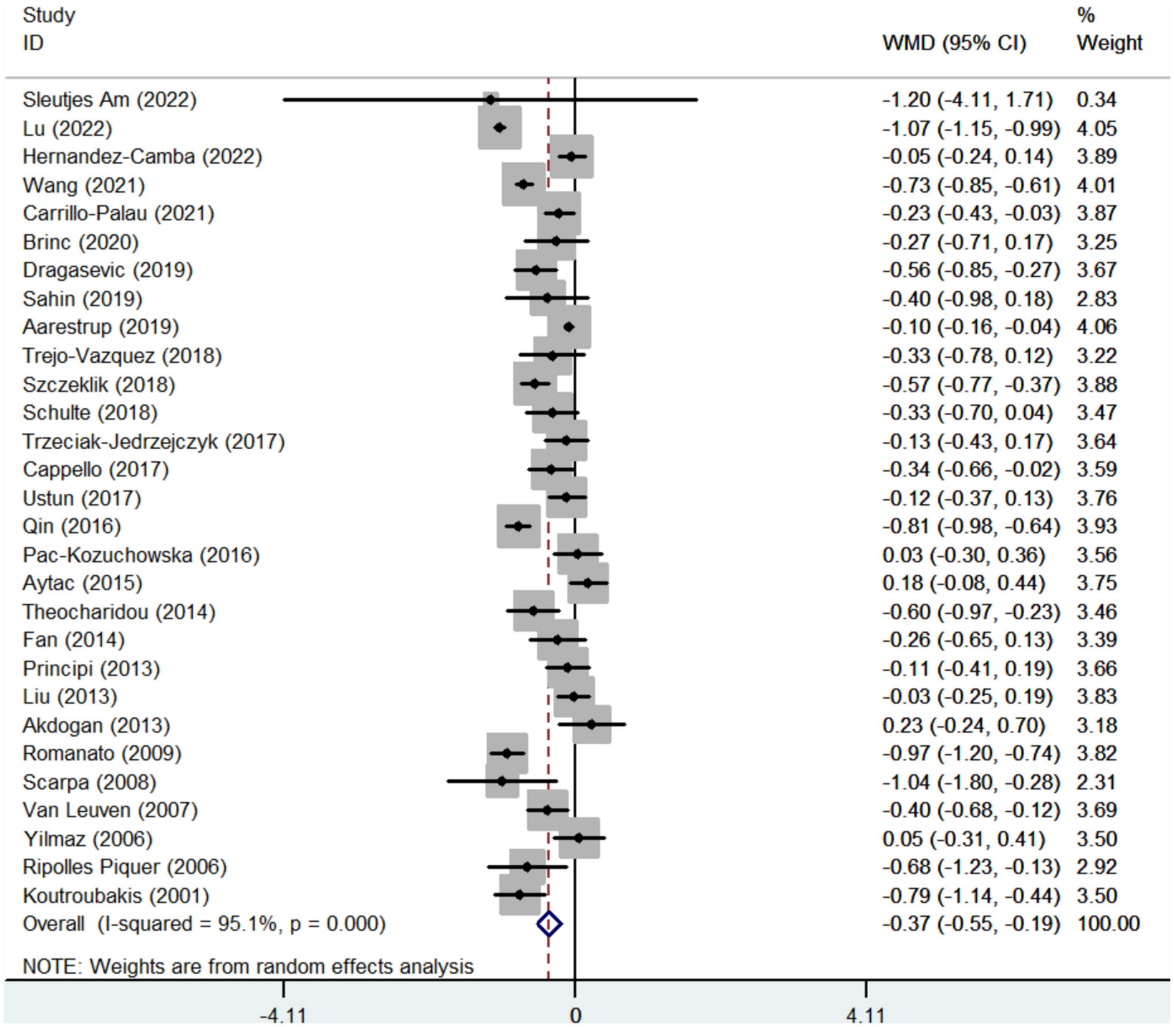
Forest plots showing the LDL- c level between IBD and healthy controls.

Twenty-nine studies reported the data regarding the LDL-c level. Meta-analysis demonstrated that IBD groups had a significantly lower level of LDL-c than healthy control groups (WMD = −0.371, 95%CI = −0.547 to −0.194, *p* < 0.001) (Figure 4). The heterogeneity was significant (*I*^2^ = 95.1%, *p* < 0.001). Sensitivity analysis and meta-regression did not find the source of heterogeneity (Supplementary Figure S1C; Supplementary Table S3).

In the subgroup analyses of IBD types, 11 and 11 studies reported the data regarding the LDL-c level in CD groups and healthy control groups, and UC groups and healthy control groups, respectively. Compared with control groups, the level of LDL-c was also significantly lower in both CD and UC groups (WMD = −0.550, 95%CI = −0.768 to −0.333, *p* < 0.001) (WMD = −0.386, 95%CI = −0.646 to −0.127, *p* = 0.003) (Supplementary Figures S2C, S4C). The heterogeneity was significant (*I*^2^ = 92.4%, *p* < 0.001) (*I*^2^ = 86.2%, *p* < 0.0001). Sensitivity analysis and meta-regression analyses did not find the source of heterogeneity (Supplementary Figures S3C, S5C; Supplementary Table S3).

#### 3.4.4. Triglyceride level

**Figure 5:**
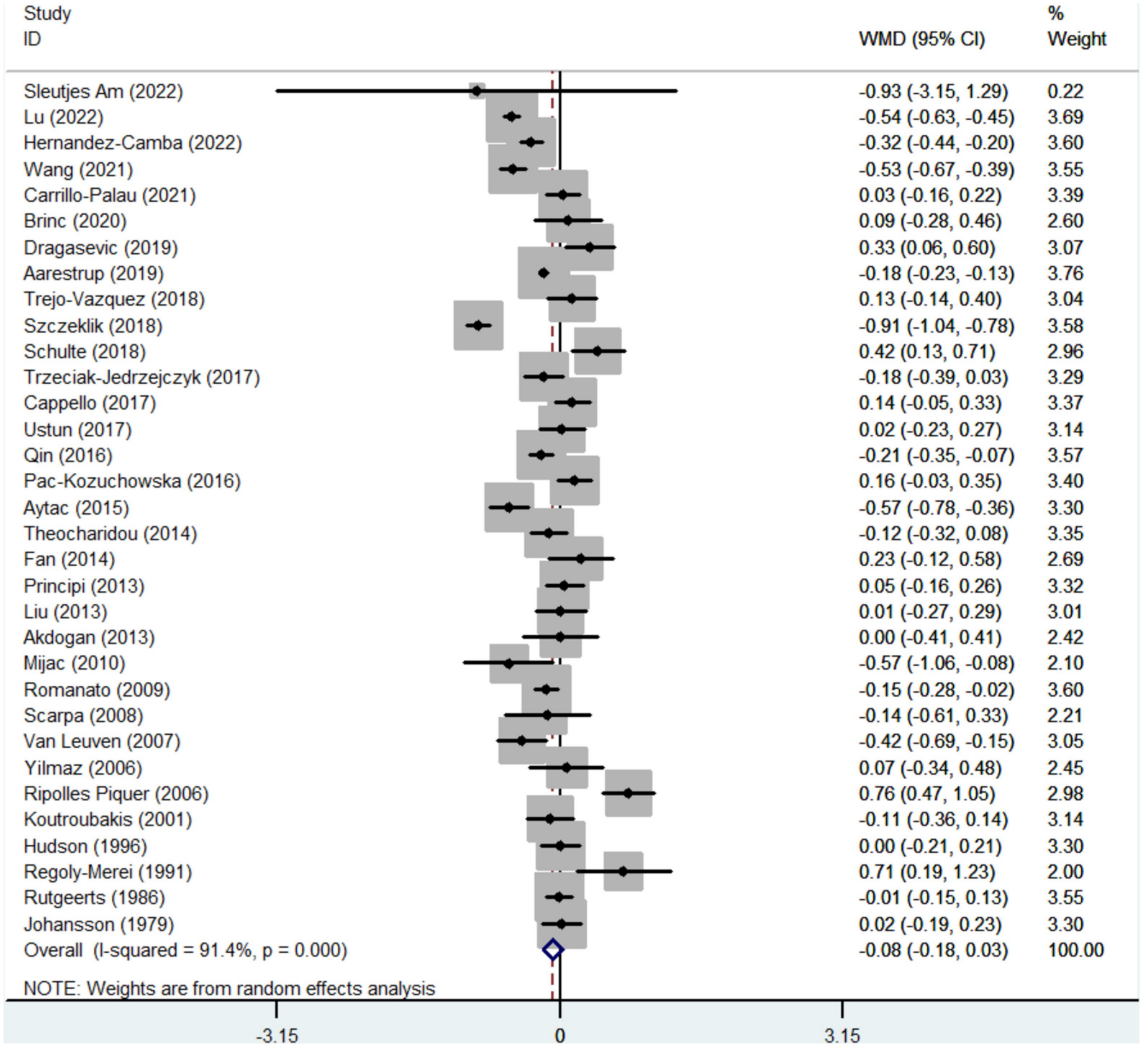
Forest plots showing the TG level between IBD and healthy controls.

Thirty-three studies reported the data regarding the TG level. Meta-analysis demonstrated that IBD groups had a lower level of TG than healthy control groups, but there was no significant difference between the two groups (WMD = −0.077, 95%CI = −0.185 to 0.031, *p* = 0.161) (Figure 5). The heterogeneity was significant (*I*^2^ = 91.4%, *p* < 0.0001). Sensitivity analysis and meta-regression did not find the source of heterogeneity (Supplementary Figure S1D; Supplementary Table S3).

In the subgroup analyses of IBD types, 11 and 11 studies reported the data regarding the TG level in CD groups and healthy control groups, and UC groups and healthy control groups, respectively. Compared with control groups, the level of TG was significantly lower in CD groups (WMD = −0263, 95%CI = −0.426 to −0.101, *p* = 0.001), but not UC groups (WMD = −0.074, 95%CI = −0.267 to 0.119, *p* = 0.452) (Supplementary Figures S2D, S4D). The heterogeneity was significant (*I*^2^ = 92.2%, *p* < 0.0001) (*I*^2^ = 82.7%, *p* < 0.0001). Sensitivity analysis and meta-regression analyses did not find the source of heterogeneity (Supplementary Figures S3D, S5D; Supplementary Table S3).

### 3.5. Meta-analysis of serum lipid levels between CD versus UC

#### 3.5.1. Total cholesterol level

**Figure 6:**
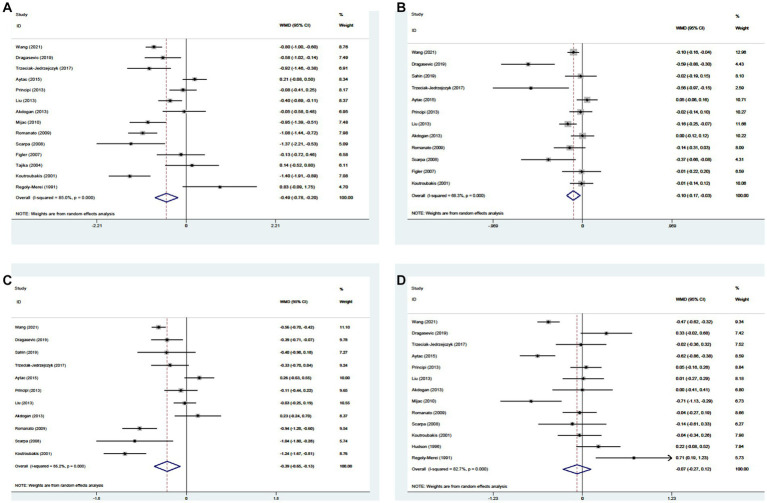
Forest plots showing the levels of TC **(A)**, HDL-c **(B)**, LDL-c **(C)**, and TG **(D)** between CD and UC.

Twenty-one studies reported the data regarding the TC level. Meta-analysis demonstrated that CD groups had a lower level of TC than UC groups (WMD = −0.349, 95%CI = −0.528 to −0.170, *p* < 0.0001) (Figure 6A). The heterogeneity was significant (*I*^2^ = 86.2%, *p* < 0.0001). Sensitivity analysis did not find the source of heterogeneity (Supplementary Figure S6A; Supplementary Table S3). Meta-regression analyses found that the source of heterogeneity might be the region and study design (Supplementary Table S3).

#### 3.5.2. High density lipoprotein cholesterol level

Fifteen studies reported the data regarding the HDL-c level. Meta-analysis demonstrated that the HDL-c level was not significantly different between CD groups and UC groups (WMD = −0.024, 95%CI = −0.068 to 0.020, *p* = 0.285) (Figure 6B). The heterogeneity was significant (*I*^2^ = 50.1%, *p* = 0.014). Sensitivity analysis did not find the source of heterogeneity (Supplementary Figure S6B). Meta-regression analyses found that the source of heterogeneity might be the public year (Supplementary Table S3).

#### 3.5.3. Low density lipoprotein cholesterol level

Thirteen studies reported the data regarding the LDL-c level. Meta-analysis demonstrated that the LDL-c level was not significantly different between CD groups and UC groups (WMD = −0.097, 95%CI = −0.297 to 0.103, *p* = 0.344) (Figure 6C). The heterogeneity was significant (*I*^2^ = 88.5%, *p* < 0.00C01). Sensitivity analysis and meta-regression analyses did not find the source of heterogeneity (Supplementary Figure S6C; Supplementary Table S3).

#### 3.5.4. Triglyceride level

Eighteen studies reported the data regarding the TG level. Meta-analysis demonstrated that the TG level was not significantly different between CD groups and UC groups (WMD = 0.014, 95%CI = −0.077 to 0.105, *p* = 0.760) (Figure 6D). The heterogeneity was significant (*I*^2^ = 78.2%, *p* < 0.0001). Sensitivity analysis and meta-regression analyses did not find the source of heterogeneity (Supplementary Figure S6D; Supplementary Table S3).

### 3.6. Meta-analysis of serum lipid levels and disease activity

#### 3.6.1. Active IBD versus inactive IBD

There were 5, 4, 4, and 4 studies reported the data regarding the TC, HDL-c, LDL-c, and TG levels, respectively. Compared with inactive IBD groups, active IBD groups had significantly lower levels of TC (WMD = -0.454, 95%CI = −0.722 to −0.187, *p* = 0.001) and LDL-c (WMD = −0.225, 95%CI = −0.445 to −0.005, *p* = 0.045), while the levels of HDL-c (WMD = −0.248, 95%CI = −0.542 to 0.047, *p* = 0.099) and TG (WMD = −0.129, 95%CI = −0.273 to 0.015, *p* = 0.080) were lower, but there were no statistically different (Table 2). There was no significant heterogeneity among studies in TC (*I*^2^ = 0%; *p* = 0.421), LDL-c (*I*^2^ = 0%; *p* = 0.740), and TG (*I*^2^ = 0%; *p* = 0.868) levels, but not HDL-c level (*I*^2^ = 87.6%; *p* < 0.0001). It is inappropriate to conduct sensitivity analysis and meta-regression analyses to explore the sources of heterogeneity since less than 10 studies were included.

**Table 2:** Meta-analyses of serum lipid levels and disease activity.

Endpoints	No. studies	Pooled proportion using random-effects model		Heterogeneity	
		WMD	*P*	*I* ^2^	*P*
**Active IBD versus inactive IBD**					
TC level	5	−0.454, 95%CI = −0.722 to −0.187	*0.001*	0%	0.421
HDL-c level	4	−0.248, 95%CI = −0.542 to 0.047	0.099	87.6%	*<0.001*
LDL-c level	4	−0.225, 95%CI = −0.445 to −0.005	*0.045*	0%	0.740
TG level	4	−0.129, 95%CI = −0.273 to 0.015	0.080	0%	0.868
**Active CD versus active UC**					
TC level	2	−0.311, 95%CI = −0.648 to 0.026	0.071	0%	0.435
HDL-c level	2	-0.075, 95%CI = −0.196 to 0.046	0.226	0%	0.891
LDL-c level	2	−0.393, 95%CI = −0.666 to −0.121	*0.005*	0.7%	0.316
TG level	2	0.077, 95%CI = −0.148 to 0.302	0.502	26.3%	0.244
**Inactive CD versus inactive UC**					
TC level	2	−0.629, 95%CI = −0.966 to −0.291	*<0.0001*	0%	0.683
HDL-c level	2	−0.059, 95%CI = −0.176 to 0.058	0.324	0%	0.378
**Mild active UC versus non-mild UC**					
TC level	2	0.462, 95%CI = 0.176 to 0.748	*0.002*	0%	0.504
LDL-c level	2	0.346, 95%CI = 0.084 to 0.609	*0.010*	0%	0.647

WMD, Weighted mean difference; CI, Confidence Interval; IBD, inflammatory bowel disease; CD, Crohn’s disease; UC, ulcerative colitis; TC, total cholesterol; HDL-c, high density lipoprotein cholesterol; LDL-c, low density lipoprotein cholesterol; TG, triglyceride.

The *p*-value was statistically significant.

#### 3.6.2. Active CD versus active UC

Two studies reported the data regarding the TC, HDL-c, LDL-c, and TG levels Compared with active UC groups, active CD groups had a significantly lower level of LDL-c (WMD = −0.393, 95%CI = −0.666 to −0.121, *p* = 0.005). Although there were no significant differences in TC (WMD = -0.311, 95%CI = −0.648 to 0.026, *p* = 0.071), HDL-c (WMD = −0.075, 95%CI = −0.196 to 0.046, *p* = 0.226), and TG (WMD =0.077, 95%CI = −0.148 to 0.302, *p* = 0.502) levels between active CD groups and active UC groups, active CD groups had lower TC and HDL-c levels, and active UC groups had a lower TG level (Table 2). There was no significant heterogeneity among studies in LDL-c (*I*^2^ = 0.7%; *p* = 0.316), TC (*I*^2^ = 0%; *p* = 0.435), HDL-c (*I*^2^ = 0%; *p* = 0.891), and TG (*I*^2^ = 26.3%; *p* = 0.244) levels. It is inappropriate to conduct sensitivity analysis and meta-regression analyses to explore the sources of heterogeneity since only two studies were included.

#### 3.6.3. Inactive CD versus inactive UC

There were 2, 2, 1, and 1 studies reported the data regarding the TC, HDL-c, LDL-c, and TG levels, respectively. Compared with inactive UC groups, inactive CD groups had a significantly lower level of TC (WMD = −0.629, 95%CI = −0.966 to −0.291, *p* < 0.0001). Although there was no significant difference in HDL-c (WMD = −0.059, 95%CI = −0.176 to 0.058, *p* = 0.324) level between inactive CD groups and inactive UC groups, inactive CD groups had a lower HDL-c level (Table 2). There was no significant heterogeneity among studies in TC (*I*^2^ = 0%; *p* = 0.683) and HDL-c (*I*^2^ = 0%; *p* = 0.378) levels. Only one study recorded data on LDL-c and TG levels, and we found that LDL-c seemed to be lower inactive CD groups (2.59 ± 0.77 mmol/L vs. 2.76 ± 0.64 mmol/L), and TG level seemed to be lower in inactive UC groups (1.01 ± 0.28 mmol/L vs. 0.91 ± 0.41 mmol/L).

#### 3.6.4. Mild active UC versus non-mild active UC

There were 2, 1, 2, and 1 studies reported the data regarding the TC, HDL-c, LDL-c, and TG levels, respectively. Compared with mild active UC groups, non-mild active UC groups had significantly lower levels of TC (WMD =0.462, 95%CI = 0.176 to 0.748, *p* = 0.002) and LDL-c (WMD =0.346, 95%CI = 0.084 to 0.609, *p* = 0.010) (Table 2). There was no significant heterogeneity among studies in TC (*I*^2^ = 0%; *p* = 0.504) and LDL-c (*I*^2^ = 0%; *p* = 0.647) levels. Only one study recorded data on HDL-c and TG levels, and we found that HDL-c level seemed to be lower in non-mild UC groups (1.23 ± 0.29 mmol/L vs. 1.05 ± 0.33 mmol/L), and TG level seemed to be no different between the two groups (1.43 ± 1.04 mmol/L vs.1.44 ± 0.98 mmol/L).

### 3.7. Publication bias

Publication bias was reported in Supplementary Table S4.

## 4. Discussion

The current systematic review and meta-analysis of 53 studies comprehensively explored the association between IBD and serum lipid levels. We found that the levels of TC, HDL-c, and LDL-c were significantly lower in IBD patients than those without. In the subgroup analyses of IBD types, we found the same findings in UC patients, but CD patients still had a significantly lower level of TG than healthy controls. In addition, we found that CD patients had a significantly lower TC level than UC patients, and active IBD and non-mild UC patients had significantly lower levels of TC and LDL-c levels than inactive IBD and mild UC patients, respectively.

Our study has several major features in the study design and statistical analysis. First, our study is the first systematic review and meta-analysis to explore the association between serum lipids and IBD. Second, subgroup analyses were planned to further explore the association between IBD types and serum lipids. Third, the selection of the population included in our meta-analysis was rational and rigorous. Specifically, we excluded studies that identified patients with comorbidities and those that specially excluded patients with dyslipidemia, which is important to eliminate the influence of these potential confounders on the reliability of our findings. Fourth, in some of the included studies, continuous data were expressed as median with range or inter quartile range. In order to perform mete-analysis, we transformed such data into means with standard deviations by Box-Cox method (27), which has been proven to be superior to all existing methods.

The association of serum lipids with IBD can be explained by the following considerations. The first one is HDL-c. Normally, Apolipoprotein AI (Apo-AI) is considered as the main apolipoprotein of HDL (67). When inflammation occurs in the body, the inflammatory factors interleukin (IL)-1, IL-6, and tumor necrosis factor-α will induce the liver to synthesize a large amount of serum amyloid A (SAA), and the SAA released into the blood quickly binds to HDL, competitively replacing Apo-AI to become the main apolipoprotein of HDL (68). SSA-containing HDL is cleared more rapidly from the circulation than normal HDL and is preferentially taken up by macrophages rather than hepatocytes, thereby decrease the HDL-c level (69, 70). Moreover, in adipose tissue, glycoproteins on the surface of adipocyte membranes can bind to SAA, causing HDL to remain in adipose tissue, reducing the concentration of plasma HDL, and ultimately leading to a lower HDL-c level in IBD patients (71). More importantly, HDL has been proven to have immunomodulatory effects (72). In cellular immunity, major histocompatibility complex (MHC) class II molecules, which play an important role in antigen presentation and signal transduction, are located in lipid-rich microdomains in antigen-presenting cells, and its number is critical for T cell activation. Lipid rafts, which are membrane microdomains containing high concentrations of cholesterol, proteins, and sphingolipids, whose functional properties depend on their lipid composition, thus depleting cholesterol from these microdomains can downregulate several signaling pathways in immune cells and disrupt antigen presentation function. It also reduces the amount of antigen required for T cell activation by concentrating MHC–peptide complexes on the surface of antigen-presenting cells (73). HDL can promote the removal of cholesterol from peripheral cells and may decrease the level of cholesterol in lipid rafts, thereby decreasing the number of MHC class II molecules and ultimately impairing T-cell activation (74). When the HDL-c level is too low to mediate immunity, there may be increased inflammation, which is why our meta-analyses found that the level of HDL-c in IBD patients was lower than in healthy controls. The second one is LDL-c. To the best of our knowledge, LDL-c is a kind of bad cholesterol, and the lower the better. However, we found that the LDL-c level was significantly lower in IBD patients than in healthy controls, and significantly lower in patients with active IBD than in patients with inactive IBD. In other diseases (i.e., COVID-19, dialysis patients, coronary heart disease, and depression), a U-shaped association between the LDL-c level and disease development and poor prognosis has been found, despite adjustment for factors such as age, nutritional status, and statin use (75–78). This suggests that LDL-c within a certain range is not associated with the occurrence and development of the disease, where excessively low or high levels may result in aggravated the disease. Therefore, we propose a reasonable hypothesis that although LDL-c is not beneficial, a low level of LDL-c in IBD patients may also mediate inflammation and promote disease progression. *In vitro* and *in vivo* experiments found that LDL can decrease the production of lipopolysaccharide (LPS)-stimulated pro-inflammatory cytokines by binding to LPS (79). LDL receptor-deficient mice can lead to increased levels of endogenous LDL-c, which can protect them from the influence of LPS and reduce the production of pro-inflammatory factors such as tumor necrosis factor and IL-1α (80). In contrast, in hypolipidemic mice, LPS induction resulted in increased mortality, which can be reversed by administering exogenous lipoproteins to raise serum lipid levels to within the physiological range (81). In addition, Coenzyme Q10, an endogenous antioxidant, is a component of LDL (82). It has been reported to inhibit the arachidonic acid metabolic pathway and the formation of various prostaglandins (83). When the level of LDL-c is too low, the level of Coenzyme Q10 may also be reduced, resulting in increased inflammation. The third one is TC. The mechanism by which inflammation lowers cholesterol levels is still unclear. Some mechanistic studies using human hepatoma HepG2 cells found that IL-1 can inhibit cholesterol synthesis and decrease cholesterol and Apo-B secretion, and IL-6 can increase cholesterol synthesis but decrease even more cholesterol secretion (84, 85). Compared with healthy individuals, there is no doubt that the levels of these inflammatory cytokines are higher in patients with IBD (44). Furthermore, during inflammation, the levels of TC and LDL-c decrease maybe due to the increase of small dense LDL-c and are more profoundly observed in diseases with more severe underlying inflammation (12). In plasma, there is active lipid exchange occurring between various lipoproteins, including TC transfer from LDL to very low-density lipoprotein (VLDL) and TG transfer from VLDL to LDL. When the level of TG in LDL increases beyond a certain threshold, LDL will be hydrolyzed by liver lipase to remove TG, resulting in smaller LDL particles and decreased TC content, forming small dense LDL-c. It is important to note that the total amount and synthesis of LDL remain unaltered in this process. Thus, the levels of LDL-c and TC decrease (86). Current studies revealed that in subtypes of LDL, small dense LDL is more susceptible to oxidation and possess pro-inflammatory effects (84, 86). The level of small dense LDL may be positively correlated with inflammation, indicating that a higher level of small dense LDL is associated with more severe inflammation (87). Admittedly, the degree of inflammation in active IBD and non-mild active UC is significantly heavier than those in patients with inactive IBD and mild active UC, respectively, so the level of small dense LDL may be higher in the active IBD and non-mild active UC. This may be one of the reasons why the levels of TC and LDL-c in active IBD and non-mild active UC are lower than those in patients with inactive IBD and mild active UC, respectively. However, due to the lack of relevant study and this is only a hypothesis based on the literature. Last, IBD is a chronic inflammatory disease involving the gastrointestinal tract, which can lead to intestinal absorption dysfunction. Therefore, the decreased levels of HDL-c, LDL-c, and TC may indicate a malnourished status in patients with IBD (12).

In our meta-analysis, we also found that the level of TC was significantly lower in CD patients than in UC patients, and the level of TG was significantly lower in CD patients than in healthy controls. A possible reason for these results is that CD more often involves in the small intestine. The terminal ileum is primarily responsible for the absorption of bile acids. When the absorption of small intestine is dysfunctional, a large amount of bile acids and cholesterol can be excreted with stools, which may decrease in lipid profiles (54). Moreover, the small intestine is also one of the main pathways for the production of TG. In small intestine, bile acids are bound to dietary triacylglycerols to facilitate their hydrolysis into free fatty acids and monoacylglycerols, which are then synthesized into TG in the endoplasmic reticulum (88). As mentioned before, CD mainly involves the small intestine, and then TG production will decrease, resulting in a lower level of TG in CD patients than in healthy controls.

The limitations need to be acknowledged. First, the definitions of disease activity and severity were not completely equal. Second, most of the included studies had a small sample size and were conducted at a single center. Third, the previous treatment strategies for patients may be inconsistent among studies, and we could not extract relevant data. Fourth, the heterogeneity among studies were significant, despite sensitivity analyses and meta-regression analyses. Fifth, there is a lack of detailed information on race or ethnicity, which may hinder the exploration of the relationship between serum lipids and IBD in different races or ethnic groups.

In conclusion, the serum lipid levels of IBD patients are lower than that of healthy controls, and active and non-mild IBD patients appear to have lower lipid levels than those in remission and mild patients, respectively. More well-designed prospective studies are needed to confirm our findings, and experimental studies are still needed to elucidate the underlying mechanisms in the future.

## Data availability statement

The original contributions presented in the study are included in the article/Supplementary material, further inquiries can be directed to the corresponding authors.

## Author contributions

HC reviewed and searched the literature, wrote the protocol, collected the data, performed the statistical analysis and quality assessment, interpreted the data, and drafted the manuscript. WL, JH, FX, and YL checked the data, discussed the findings, and gave critical comments. HS and LZ conceived the work, reviewed, searched the literature, wrote the protocol, performed the statistical analysis, interpreted the data, and revised the manuscript. All authors contributed to the article and approved the submitted version.

## Funding

This work was supported by the Jiangsu Provincial Medical Innovation Center.

## Conflict of interest

The authors declare that the research was conducted in the absence of any commercial or financial relationships that could be construed as a potential conflict of interest.

## Publisher’s note

All claims expressed in this article are solely those of the authors and do not necessarily represent those of their affiliated organizations, or those of the publisher, the editors and the reviewers. Any product that may be evaluated in this article, or claim that may be made by its manufacturer, is not guaranteed or endorsed by the publisher.

## Abbreviations

IBD: inflammatory bowel disease
CD: Crohn’s disease
UC: ulcerative colitis
TC: total cholesterol
HDL-c: high density lipoprotein
LDL-c: low density lipoprotein cholesterol
TG: triglyceride
NOS: Newcastle-Ottawa scale
AHRQ: Agency for Healthcare Research and Quality
WMD: weight mean difference
CIs: confidence intervals
IL: inflammatory factors interleukin
Apo-AI: apolipoprotein AI
SAA: serum amyloid A
MHC: major histocompatibility complex
LPS: lipopolysaccharide
VLDL: very low density lipoprotein

## References

[1] Bisgaard TH Allin KH Keefer L Ananthakrishnan AN Jess T. Depression and anxiety in inflammatory bowel disease: epidemiology, mechanisms and treatment. Nat Rev Gastroenterol Hepatol. (2022) 19:717–26. doi: 10.1038/s41575-022-00634-6, PMID: 35732730

[2] Ng SC Shi HY Hamidi N Underwood FE Tang W Benchimol EI. Worldwide incidence and prevalence of inflammatory bowel disease in the 21st century: a systematic review of population-based studies. Lancet. (2017) 390:2769–78. doi: 10.1016/S0140-6736(17)32448-0, PMID: 29050646

[3] Adolph TE Meyer M Schwärzler J Mayr L Grabherr F Tilg H. The metabolic nature of inflammatory bowel diseases. Nat Rev Gastroenterol Hepatol. (2022) 19:753–67. doi: 10.1038/s41575-022-00658-y 35906289

[4] Song Y Zhao Y Ma Y Wang Z Rong L Wang B. Biological functions of NLRP3 inflammasome: a therapeutic target in inflammatory bowel disease. Cytokine Growth Factor Rev. (2021) 60:61–75. doi: 10.1016/j.cytogfr.2021.03.003, PMID: 33773897

[5] Sadat Masjedi M Mohammadi Pour P Shokoohinia Y Asgary S. Effects of flaxseed on blood lipids in healthy and dyslipidemic subjects: a systematic review and Meta-analysis of randomized controlled trials. Curr Probl Cardiol. (2022) 47:100931. doi: 10.1016/j.cpcardiol.2021.100931 34384619

[6] de Carvalho JF Bonfá E Borba EF. Systemic lupus erythematosus and “lupus dyslipoproteinemia”. Autoimmun Rev. (2008) 7:246–50. doi: 10.1016/j.autrev.2007.11.016 18190886

[7] Kim SY Yu M Morin EE Kang J Kaplan MJ Schwendeman A. High-density lipoprotein in lupus: disease biomarkers and potential therapeutic strategy. Arthritis Rheumatol. (2020) 72:20–30. doi: 10.1002/art.41059, PMID: 31350818 PMC6935404

[8] Borba EF Carvalho JF Bonfá E. Mechanisms of dyslipoproteinemias in systemic lupus erythematosus. Clin Dev Immunol. (2006) 13:203–8. doi: 10.1080/17402520600876945, PMID: 17162363 PMC2270754

[9] Coelewij L Waddington KE Robinson GA Chocano E McDonnell T Farinha F. Serum metabolomic signatures can predict subclinical atherosclerosis in patients with systemic lupus erythematosus. Arterioscler Thromb Vasc Biol. (2021) 41:1446–58. doi: 10.1161/ATVBAHA.120.315321, PMID: 33535791 PMC7610443

[10] Lu L Hu C Zhao Y He L Zhou J Li H. Shotgun Lipidomics revealed altered profiles of serum lipids in systemic lupus erythematosus closely associated with disease activity. Biomol Ther. (2018) 8:105. doi: 10.3390/biom8040105, PMID: 30282943 PMC6315961

[11] Liu Z Tang H Liang H Bai X Zhang H Yang H. Dyslipidaemia is associated with severe disease activity and poor prognosis in ulcerative colitis: a retrospective cohort study in China. Nutrients. (2022) 14:3040. doi: 10.3390/nu14153040 35893893 PMC9330762

[12] Soh H Im JP Han K Park S Hong SW Moon JM. Crohn's disease and ulcerative colitis are associated with different lipid profile disorders: a nationwide population-based study. Aliment Pharmacol Ther. (2020) 51:446–56. doi: 10.1111/apt.15562, PMID: 31691306

[13] Wang D Zhao XJ Cui XF Li LZ Zhang HJ. Correlation of serum lipid profile and disease activity in patients with inflammatory bowel disease. Zhonghua Nei Ke Za Zhi. (2021) 60:834–6. doi: 10.3760/cma.j.cn112138-20200930-00847, PMID: 34445822

[14] Lu J Yu F Huang J Yu H Li F Le Z. Hypocholesterolemia and inflammatory biomarkers act as predictors of severe vitamin D deficiency in patients with Crohn's disease: a clinical analysis of 862 patients in China. Front Nutr. (2022) 9:806887. doi: 10.3389/fnut.2022.806887, PMID: 35495921 PMC9043686

[15] Schulte DM Paulsen K Türk K Brandt B Freitag-Wolf S Hagen I. Small dense LDL cholesterol in human subjects with different chronic inflammatory diseases. Nutr Metab Cardiovasc Dis. (2018) 28:1100–5. doi: 10.1016/j.numecd.2018.06.022, PMID: 30143407

[16] Ripollés Piquer B Nazih H Bourreille A Segain JP Huvelin JM Galmiche JP. Altered lipid, apolipoprotein, and lipoprotein profiles in inflammatory bowel disease: consequences on the cholesterol efflux capacity of serum using Fu5AH cell system. Metabolism. (2006) 55:980–8. doi: 10.1016/j.metabol.2006.03.006 16784973

[17] Hernández-Camba A Carrillo-Palau M Ramos L de Armas-Rillo L Vela M Arranz L. Apolipoprotein C3 is downregulated in patients with inflammatory bowel disease. Clin Transl Gastroenterol. (2022) 13:e00500. doi: 10.14309/ctg.0000000000000500, PMID: 35584319 PMC9236603

[18] Mańkowska-Wierzbicka D Karczewski J Swora-Cwynar E Dobrowolska A Stelmach-Mardas M. The clinical importance of 21-day combined parenteral and enteral nutrition in active inflammatory bowel disease patients. Nutrients. (2019) 11:2246. doi: 10.3390/nu11092246, PMID: 31540473 PMC6770879

[19] Qin G Tu J Liu L Luo L Wu J Tao L. Serum albumin and C-reactive protein/albumin ratio are useful biomarkers of Crohn’s disease activity. Med Sci Monit. (2016) 22:4393–400. doi: 10.12659/MSM.897460, PMID: 27848931 PMC12574461

[20] Aytac E Buyuktas D Baysal B Atar M Yildiz M Baca B. Visual evoked potentials and pulse wave velocity in inflammatory bowel disease. Turk J Gastroenterol. (2015) 26:15–9. doi: 10.5152/tjg.2015.4349, PMID: 25698265

[21] Hrabovský V Zadák Z Bláha V Hyšpler R Karlík T Martínek A. Cholesterol metabolism in active Crohn's disease. Wien Klin Wochenschr. (2009) 121:270–5. doi: 10.1007/s00508-009-1150-6 19562284

[22] Levy E Rizwan Y Thibault L Lepage G Brunet S Bouthillier L. Altered lipid profile, lipoprotein composition, and oxidant and antioxidant status in pediatric Crohn disease. Am J Clin Nutr. (2000) 71:807–15. doi: 10.1093/ajcn/71.3.807, PMID: 10702177

[23] De Fatima AE Bodanese LC. Evaluation of lipid profile in patients with inflammatory bowel disease. Sci Med. (2016) 26:1–9. doi: 10.15448/1980-6108.2016.2.22964

[24] Van Leuven SI Hezemans R Levels JH Snoek S Stokkers PC Hovingh GK. Enhanced atherogenesis and altered high density lipoprotein in patients with Crohn's disease. J Lipid Res. (2007) 48:2640–6. doi: 10.1194/jlr.M700176-JLR200, PMID: 17890779

[25] Li X Liu X Song Y. Expression and clinical significance of NLRP1 and NLRP3 in colonic tissues of patients with ulcerative colitis. J Xi'an Jiaotong Univ. (2021) 42:75–80, 112. doi: 10.7652/jdyxb202101014

[26] Liu W Liu X Yu X Tao Y Yang G. Correlation between active ulcerative colitis and protein, lipid metabolisms. Chin Jo Gastroenterol. (2013) 18:738–40. doi: 10.3969/j.issn.1008-7125.2013.12.008

[27] McGrath S Zhao X Steele R Thombs BD Benedetti A. Estimating the sample mean and standard deviation from commonly reported quantiles in meta-analysis. Stat Methods Med Res. (2020) 29:2520–37. doi: 10.1177/0962280219889080, PMID: 32292115 PMC7390706

[28] Sleutjes Am J Roeters van Lennep JE Kavousi M Aribas E Van Der Woude CJ De Vries AC. Increased risk of cardiovascular disease and high risk profiles compatible with metabolic syndrome in patients with inflammatory bowel disease: a cross-sectional analysis of matched cohorts. J Crohn's Colitis. (2022) 16:i586. doi: 10.1093/ecco-jcc/jjab232.805

[29] Carrillo-Palau M Hernández-Camba A Hernández Alvarez-Buylla N Ramos L Alonso-Abreu I Hernández-Pérez A. Insulin resistance is not increased in inflammatory bowel disease patients but is related to non-alcoholic fatty liver disease. J Clin Med. (2021) 10:3062. doi: 10.3390/jcm10143062, PMID: 34300227 PMC8304915

[30] Vrdoljak J Vilović M Živković PM Tadin Hadjina I Rušić D Bukić J. Mediterranean diet adherence and dietary attitudes in patients with inflammatory bowel disease. Nutrients. (2020) 12:1–14. doi: 10.3390/nu12113429 PMC7695291 33171662

[31] Brnić D Martinovic D Zivkovic PM Tokic D Tadin Hadjina I Rusic D. Serum adropin levels are reduced in patients with inflammatory bowel diseases. Sci Rep. (2020) 10:9264. doi: 10.1038/s41598-020-66254-9, PMID: 32518265 PMC7283308

[32] Dragasevic S Stankovic B Kotur N Sokic-Milutinovic A Milovanovic T Lukic S. Metabolic syndrome in inflammatory bowel disease: association with genetic markers of obesity and inflammation. Metab Syndr Relat Disord. (2019) 18:31–8. doi: 10.1089/met.2019.0090, PMID: 31750766

[33] Sahin M Bobusoglu O Yetim A Ates F. Paraoxonase-1 and arylesterase levels in patients with ulcerative colitis. Arab J Gastroenterol. (2019) 20:14–8. doi: 10.1016/j.ajg.2019.01.009, PMID: 30745012

[34] Qiao Y Zhou M Shen J Ran Z. Bioelectrical impedance analysis for body composition and nutritional status in hospitalized patients with inflammatory bowel disease. Chin J Gastroenterol. (2019) 24:5–9.

[35] Iwakawa H Fukui T Fukuwatari T Bamba S Sasaki M Tsujikawa T. Blood concentrations and renal clearance of water-soluble vitamins in outpatients with ulcerative colitis. Biomed Rep. (2019) 10:202–10. doi: 10.3892/br.2019.1191, PMID: 30906550 PMC6403479

[36] Kang EA Han K Chun J Soh H Park S Im JP. Increased risk of diabetes in inflammatory bowel disease patients: a Nationwide population-based study in Korea. J Clin Med. (2019) 8:343. doi: 10.3390/jcm8030343, PMID: 30862129 PMC6463263

[37] Aarestrup J Jess T Kobylecki CJ Nordestgaard BG Allin KH. Cardiovascular risk profile among patients with inflammatory bowel disease: a population-based study of more than 100 000 individuals. J Crohns Colitis. (2019) 13:319–23. doi: 10.1093/ecco-jcc/jjy164, PMID: 30321330

[38] Trejo-Vazquez F Garza-Veloz I Villela-Ramirez GA Ortiz-Castro Y Mauricio-Saucedo P Cardenas-Vargas E. Positive association between leptin serum levels and disease activity on endoscopy in inflammatory bowel disease: a case-control study. Exp Ther Med. (2018) 15:3336–44. doi: 10.3892/etm.2018.5835, PMID: 29545852 PMC5841033

[39] Szczeklik K Mach T Cibor D Owczarek D Sapa J Papież M. Correlation of Paraoxonase-1 with the severity of Crohn's disease. Molecules. (2018) 23:2063. doi: 10.3390/molecules23102603 30314292 PMC6222603

[40] Grzybowska-Chlebowczyk U Wysocka-Wojakiewicz P Jasielska M Cukrowska B Więcek S Kniażewska M. Oxidative and antioxidative stress status in children with inflammatory bowel disease as a result of a chronic inflammatory process. Mediat Inflamm. (2018) 2018:1–7. doi: 10.1155/2018/4120973 PMC6079420 30116148

[41] Trzeciak-Jędrzejczyk A Makosiej R Kolejwa M Głowacka E Czkwianianc E. The role of adhesion molecules in inflammatory bowel disease in children. Assessment of the possible risk of cardiovascular complications. Przeglad Gastroenterol. (2017) 12:181–5. doi: 10.5114/pg.2017.70480, PMID: 29123578 PMC5672715

[42] Cappello M Licata A Calvaruso V Bravatà I Aiello A Torres D. Increased expression of markers of early atherosclerosis in patients with inflammatory bowel disease. Eur J Intern Med. (2017) 37:83–9. doi: 10.1016/j.ejim.2016.10.004, PMID: 27773555

[43] Üstün Y Kilincalp S Çoban Ş Coşkun Y Yüksel İ Ongun A. Evaluation of early atherosclerosis markers in patients with inflammatory bowel disease. Med Sci Monit. (2016) 22:3943–50. doi: 10.12659/MSM.898160, PMID: 27773920 PMC5094468

[44] Pac-Kożuchowska E Krawiec P Mroczkowska-Juchkiewicz A Pawłowska-Kamieniak A Kominek K. Inflammatory and lipid-associated markers of cardiovascular diseases in children with first exacerbation of inflammatory bowel disease. Med Sci Monit. (2016) 22:1534–9. doi: 10.12659/MSM.896116, PMID: 27150426 PMC4917334

[45] Koutroumpakis E Ramos-Rivers C Regueiro M Hashash JG Barrie A Swoger J. Association between long-term lipid profiles and disease severity in a large cohort of patients with inflammatory bowel disease. Dig Dis Sci. (2016) 61:865–71. doi: 10.1007/s10620-015-3932-1, PMID: 26514677

[46] Aguilar-Tablada TC Navarro-Alarcón M Granados JQ Sánchez CS Rufián-Henares JÁ Nogueras-Lopez F. Ulcerative colitis and Crohn’s disease are associated with decreased serum selenium concentrations and increased cardiovascular risk. Nutrients. (2016) 8:780. doi: 10.3390/nu8120780 27916926 PMC5188435

[47] Wada Y Hisamatsu T Naganuma M Matsuoka K Okamoto S Inoue N. Risk factors for decreased bone mineral density in inflammatory bowel disease: a cross-sectional study. Clin Nutr. (2015) 34:1202–9. doi: 10.1016/j.clnu.2015.01.003, PMID: 25618799

[48] Theocharidou E Tellis CC Mavroudi M Soufleris K Gossios TD Giouleme O. Lipoprotein-associated phospholipase A2 and arterial stiffness evaluation in patients with inflammatory bowel diseases. J Crohns Colitis. (2014) 8:936–44. doi: 10.1016/j.crohns.2014.01.016, PMID: 24529818

[49] Fan F Galvin A Fang L White DA Moore XL Sparrow M. Comparison of inflammation, arterial stiffness and traditional cardiovascular risk factors between rheumatoid arthritis and inflammatory bowel disease. J Inflamm. (2014) 11:29. doi: 10.1186/s12950-014-0029-0 PMC4203921 25337037

[50] Principi M Mastrolonardo M Scicchitano P Gesualdo M Sassara M Guida P. Endothelial function and cardiovascular risk in active inflammatory bowel diseases. J Crohns Colitis. (2013) 7:e427–33. doi: 10.1016/j.crohns.2013.02.001, PMID: 23473915

[51] Akdoğan RA Durakoğlugil ME Kocaman SA Çiçek Y Durakoğlugil T Ergül E. Increased pulse wave velocity and carotid intima-media thickness in patients with ulcerative colitis. Dig Dis Sci. (2013) 58:2293–300. doi: 10.1007/s10620-013-2634-9, PMID: 23508984

[52] Yorulmaz E Adali G Yorulmaz H Ulasoglu C Tasan G Tuncer I. Metabolic syndrome frequency in inflammatory bowel diseases. Saudi J Gastroenterol. (2011) 17:376–82. doi: 10.4103/1319-3767.87177, PMID: 22064334 PMC3221110

[53] Kuwabara A Nakase H Tsuji H Shide K Chiba T Inagaki N. Fat restriction is associated with impaired quality of life in patients with ulcerative colitis and Crohn's disease. Ulcers. (2011) 2011:1–5. doi: 10.1155/2011/594532

[54] Sappati Biyyani RSR Putka BS Mullen KD. Dyslipidemia and lipoprotein profiles in patients with inflammatory bowel disease. J Clin Lipidol. (2010) 4:478–82. doi: 10.1016/j.jacl.2010.08.021, PMID: 21122694

[55] Mijac DD Janković GL Jorga J Krstić MN. Nutritional status in patients with active inflammatory bowel disease: prevalence of malnutrition and methods for routine nutritional assessment. Eur J Intern Med. (2010) 21:315–9. doi: 10.1016/j.ejim.2010.04.012, PMID: 20603043

[56] ROMANATO G SCARPA M ANGRIMAN I FAGGIAN D RUFFOLO C MARIN R. Plasma lipids and inflammation in active inflammatory bowel diseases. Aliment Pharmacol Ther. (2009) 29:298–307. doi: 10.1111/j.1365-2036.2008.03886.x 19035968

[57] Scarpa M Romanato G Manzato E Ruffolo C Marin R Basato S. Restorative proctocolectomy for ulcerative colitis: impact on lipid metabolism and adipose tissue and serum fatty acids. J Gastrointest Surg. (2008) 12:279–87. doi: 10.1007/s11605-007-0380-z, PMID: 17955308

[58] Figler M Gasztonyi B Cseh J Horváth G Kisbenedek AG Bokor S. Association of n-3 and n-6 long-chain polyunsaturated fatty acids in plasma lipid classes with inflammatory bowel diseases. Br J Nutr. (2007) 97:1154–61. doi: 10.1017/S0007114507682956, PMID: 17381967

[59] Yılmaz S Bayan K Tüzün Y Batun S Altıntaş A. A comprehensive analysis of 12 thrombophilic mutations and related parameters in patients with inflammatory bowel disease: data from Turkey. J Thromb Thrombolysis. (2006) 22:205–12. doi: 10.1007/s11239-006-9032-5, PMID: 17111197

[60] Tajika M Matsuura A Nakamura T Suzuki T Sawaki A Kato T. Risk factors for vitamin D deficiency in patients with Crohn's disease. J Gastroenterol. (2004) 39:527–33. doi: 10.1007/s00535-003-1338-x 15235869

[61] Koutroubakis IE Malliaraki N Vardas E Ganotakis E Margioris AN Manousos ON. Increased levels of lipoprotein (a) in Crohn's disease: a relation to thrombosis? Eur J Gastroenterol Hepatol. (2001) 13:1415–9. doi: 10.1097/00042737-200112000-00004, PMID: 11742189

[62] Hudson M Chitolie A Hutton RA Smith MS Pounder RE Wakefield AJ. Thrombotic vascular risk factors in inflammatory bowel disease. Gut. (1996) 38:733–7. doi: 10.1136/gut.38.5.733, PMID: 8707120 PMC1383156

[63] Hakala K Vuoristo M Miettinen TA. Serum cholestanol, cholesterol precursors and plant sterols in different inflammatory bowel diseases. Digestion. (1996) 57:83–9. doi: 10.1159/000201318, PMID: 8786005

[64] Regöly-Mérei A Ferencz A Frenkl R Gergely A Zajkás G Antal M. Effect of fat and retinol loading on serum triglyceride and retinol levels in patients with ulcerative colitis. Nahrung. (1991) 35:21–6. doi: 10.1002/food.19910350106, PMID: 1865886

[65] Rutgeerts P Ghoos Y Vantrappen G Fevery J. Biliary lipid composition in patients with nonoperated Crohn's disease. Dig Dis Sci. (1986) 31:27–32. doi: 10.1007/BF01347906 3940821

[66] Johansson C Rössner S Walldius G Kollberg B. Dyslipoproteinaemia after ileal resection in Crohn's disease. Digestion. (1979) 19:77–85. doi: 10.1159/000198327, PMID: 225234

[67] Kisilevsky R Manley PN. Acute-phase serum amyloid a: perspectives on its physiological and pathological roles. Amyloid. (2012) 19:5–14. doi: 10.3109/13506129.2011.654294, PMID: 22320226

[68] Webb NR. High-density lipoproteins and serum amyloid a (SAA). Curr Atheroscler Rep. (2021) 23:7. doi: 10.1007/s11883-020-00901-4, PMID: 33447953 PMC7808882

[69] Banka CL Yuan T de Beer MC Kindy M Curtiss LK de Beer FC. Serum amyloid a (SAA): influence on HDL-mediated cellular cholesterol efflux. J Lipid Res. (1995) 36:1058–65. doi: 10.1016/S0022-2275(20)39863-1, PMID: 7658153

[70] Artl A Marsche G Pussinen P Knipping G Sattler W Malle E. Impaired capacity of acute-phase high density lipoprotein particles to deliver cholesteryl ester to the human HUH-7 hepatoma cell line. Int J Biochem Cell Biol. (2002) 34:370–81. doi: 10.1016/S1357-2725(01)00132-7, PMID: 11854036

[71] Han CY Tang C Guevara ME Wei H Wietecha T Shao B. Serum amyloid a impairs the anti-inflammatory properties of HDL. J Clin Invest. (2016) 126:266–81. doi: 10.1172/JCI83475, PMID: 26642365 PMC4701569

[72] Catapano AL Pirillo A Bonacina F Norata GD. HDL in innate and adaptive immunity. Cardiovasc Res. (2014) 103:372–83. doi: 10.1093/cvr/cvu150, PMID: 24935428

[73] Anderson HA Roche PA. MHC class II association with lipid rafts on the antigen presenting cell surface. Biochim Biophys Acta. (2015) 1853:775–80. doi: 10.1016/j.bbamcr.2014.09.019, PMID: 25261705 PMC4344880

[74] Norata GD Pirillo A Ammirati E Catapano AL. Emerging role of high density lipoproteins as a player in the immune system. Atherosclerosis. (2012) 220:11–21. doi: 10.1016/j.atherosclerosis.2011.06.045, PMID: 21783193

[75] Johannesen CDL Langsted A Mortensen MB Nordestgaard BG. Association between low density lipoprotein and all cause and cause specific mortality in Denmark: prospective cohort study. BMJ. (2021) 372:n422. doi: 10.1136/bmj.n422 33293274 PMC7722479

[76] Gong J Chen Y Jie Y Tan M Jiang Z Yuan L. U-shaped relationship of low-density lipoprotein cholesterol with risk of severe COVID-19 from a Multicenter pooled analysis. Front Cardiovasc Med. (2021) 8:604736. doi: 10.3389/fcvm.2021.604736, PMID: 34504873 PMC8421675

[77] Wu X Zhou L Zhan X Wen Y Wang X Feng X. Low-density lipoprotein cholesterol and mortality in peritoneal Dialysis. Front Nutr. (2022) 9:910348. doi: 10.3389/fnut.2022.910348, PMID: 35938138 PMC9351358

[78] Tedders SH Fokong KD McKenzie LE Wesley C Yu L Zhang J. Low cholesterol is associated with depression among US household population. J Affect Disord. (2011) 135:115–21. doi: 10.1016/j.jad.2011.06.045, PMID: 21802743

[79] Feng Q Wei WQ Chaugai S Leon BGC Mosley JD Leon DAC. Association between low-density lipoprotein cholesterol Levels and risk for Sepsis among patients admitted to the hospital with infection. JAMA Netw Open. (2019) 2:e187223. doi: 10.1001/jamanetworkopen.2018.7223, PMID: 30657536 PMC6447031

[80] Netea MG Demacker PN Kullberg BJ Boerman OC Verschueren I Stalenhoef AF. Low-density lipoprotein receptor-deficient mice are protected against lethal endotoxemia and severe gram-negative infections. J Clin Invest. (1996) 97:1366–72. doi: 10.1172/JCI118556 8617867 PMC507194

[81] Feingold KR Funk JL Moser AH Shigenaga JK Rapp JH Grunfeld C. Role for circulating lipoproteins in protection from endotoxin toxicity. Infect Immun. (1995) 63:2041–6. doi: 10.1128/iai.63.5.2041-2046.1995, PMID: 7729918 PMC173262

[82] Pallotti F Bergamini C Lamperti C Fato R. The roles of coenzyme Q in disease: direct and indirect involvement in cellular functions. Int J Mol Sci. (2021) 23:128. doi: 10.3390/ijms23010128, PMID: 35008564 PMC8745647

[83] Lelli JL Drongowski RA Gastman B Remick DG Coran AG. Effects of coenzyme Q10 on the mediator cascade of sepsis. Circ Shock. (1993) 39:178–87. PMID: 8453741

[84] Khovidhunkit W Kim MS Memon RA Shigenaga JK Moser AH Feingold KR. Effects of infection and inflammation on lipid and lipoprotein metabolism: mechanisms and consequences to the host. J Lipid Res. (2004) 45:1169–96. doi: 10.1194/jlr.R300019-JLR200, PMID: 15102878

[85] Ettinger WH Varma VK Sorci-Thomas M Parks JS Sigmon RC Smith TK. Cytokines decrease apolipoprotein accumulation in medium from hep G2 cells. Arterioscler Thromb. (1994) 14:8–13. doi: 10.1161/01.ATV.14.1.8, PMID: 8274481

[86] Jin X Yang S Lu J Wu M. Small, dense low-density lipoprotein-cholesterol and atherosclerosis: relationship and therapeutic strategies. Front Cardiovasc Med. (2021) 8:804214. doi: 10.3389/fcvm.2021.804214 35224026 PMC8866335

[87] Feingold KR Grunfeld C. (2000) The effect of inflammation and infection on lipids and lipoproteins. In: Feingold KR Anawalt B Blackman MR Boyce A Chrousos G Corpas E. (Eds.) Endotext. South Dartmouth, MA: MDText.com.

[88] Pan X Hussain MM. Gut triglyceride production. Biochim Biophys Acta. (2012) 1821:727–35. doi: 10.1016/j.bbalip.2011.09.013, PMID: 21989069 PMC3319358

