# Supplementary material for: Association of serum lipids with inflammatory bowel disease: a systematic review and meta-analysis
Source: Front Med (Lausanne). 2023 Aug 24;10:1198988. doi: 10.3389/fmed.2023.1198988 (PMC10484721; doi:10.3389/fmed.2023.1198988)
Supplement: Supplementary file 1 [file Table_1.docx]

| **Supplementary Table S1. Quality of case-control and cohort studies.** | | | | | | | | | | |
| --- | --- | --- | --- | --- | --- | --- | --- | --- | --- | --- |
| **First author (year)** | **Quality score** | **Selection (☆☆☆☆)** | | | | **Comparability (☆☆)** | | **Outcome (☆☆☆)** | | |
| Mankowska-Wierzbicka (2019) | 7 | ☆ | ☆ | ☆ | / | ☆ | / | ☆ | / | ☆ |
| Ae Kang  (2019) | 8 | ☆ | ☆ | ☆ | / | ☆ | ☆ | ☆ | ☆ | ☆ |
| Aarestrup (2019) | 7 | ☆ | ☆ | ☆ | / | ☆ | / | ☆ | ☆ | ☆ |
| Trejo-Vazquez (2018) | 6 | / | ☆ | / | ☆ | ☆ | / | ☆ | ☆ | ☆ |
| Szczeklik (2018) | 5 | / | ☆ | / | ☆ | / | / | ☆ | ☆ | ☆ |
| Trzeciak-Jedrzejczyk (2017) | 5 | / | ☆ | / | ☆ | ☆ | / | ☆ | ☆ | ☆ |
| Cappello (2017) | 7 | / | ☆ | / | ☆ | ☆ | ☆ | ☆ | ☆ | ☆ |
| Ustun (2016) | 6 | / | ☆ | / | ☆ | ☆ | / | ☆ | ☆ | ☆ |
| Qin (2016) | 7 | / | ☆ | / | ☆ | ☆ | ☆ | ☆ | ☆ | ☆ |
| Pac-Kozuchowska (2016) | 7 | ☆ | ☆ | / | ☆ | ☆ | / | ☆ | ☆ | ☆ |
| Koutroumpakis (2016) | 7 | ☆ | ☆ | ☆ | / | ☆ | / | ☆ | ☆ | ☆ |
| Aguilar-Tablada (2016) | 8 | ☆ | ☆ | ☆ | ☆ | ☆ | / | ☆ | ☆ | ☆ |
| Aytac (2015) | 7 | ☆ | ☆ | / | ☆ | ☆ | / | ☆ | ☆ | ☆ |
| Theocharidou (2014) | 7 | ☆ | ☆ | / | ☆ | ☆ | / | ☆ | ☆ | ☆ |
| Fan (2014) | 6 | / | ☆ | / | ☆ | ☆ | / | ☆ | ☆ | ☆ |
| Principi (2013) | 8 | ☆ | ☆ | ☆ | ☆ | ☆ | / | ☆ | ☆ | ☆ |
| Hrabovsky (2009) | 7 | ☆ | ☆ | / | ☆ | ☆ | / | ☆ | ☆ | ☆ |
| Scarpa (2008) | 7 | / | ☆ | / | ☆ | ☆ | ☆ | ☆ | ☆ | ☆ |
| Van Leuven (2007) | 6 | / | ☆ | / | ☆ | ☆ | / | ☆ | ☆ | ☆ |
| Yılmaz (2006) | 7 | ☆ | ☆ | / | ☆ | ☆ | / | ☆ | ☆ | ☆ |
| Ripolles Piquer (2006) | 7 | ☆ | ☆ | / | ☆ | ☆ | / | ☆ | ☆ | ☆ |
| Koutroubakis (2001) | 7 | ☆ | ☆ | / | ☆ | ☆ | / | ☆ | ☆ | ☆ |
| Levy (2000) | 7 | ☆ | ☆ | / | ☆ | ☆ | / | ☆ | ☆ | ☆ |
| Hudson (1996) | 7 | ☆ | ☆ | / | ☆ | ☆ | / | ☆ | ☆ | ☆ |
| Hakala (1996) | 7 | ☆ | ☆ | / | ☆ | ☆ | / | ☆ | ☆ | ☆ |
| Regoly-Merei (1991) | 5 | / | ☆ | / | ☆ | ☆ | / | ☆ | ☆ | ☆ |
| Rutgeerts (1986) | 6 | / | ☆ | / | ☆ | ☆ | / | ☆ | ☆ | ☆ |
| Johansson (1979) | 8 | ☆ | ☆ | / | ☆ | ☆ | ☆ | ☆ | ☆ | ☆ |
| **Notes:** / indicates no star. | | | | | | | | | | |
