# Supplementary material for: Association of serum lipids with inflammatory bowel disease: a systematic review and meta-analysis
Source: Front Med (Lausanne). 2023 Aug 24;10:1198988. doi: 10.3389/fmed.2023.1198988 (PMC10484721; doi:10.3389/fmed.2023.1198988)
Supplement: Supplementary file 2 [file Table_2.docx]

| **Supplementary Table S2. Quality of cross-sectional studies.** | | | | | | | | | | |  |  |
| --- | --- | --- | --- | --- | --- | --- | --- | --- | --- | --- | --- | --- |
| **First author (year)** | **Q1** | **Q2** | **Q3** | **Q4** | **Q5** | **Q6** | **Q7** | **Q8** | **Q9** | **Q10** | **Q11** | **Total** |
| Sleutjes Am (2022) | Yes | Yes | No | Yes | Yes | No | No | Yes | Unclear | Yes | Unclear | 6 |
| Lu (2022) | Yes | Yes | Yes | Yes | Yes | No | Yes | Yes | Unclear | Yes | Unclear | 8 |
| Hernandez-Camba (2022) | Yes | Yes | No | No | Yes | No | Yes | Yes | Unclear | Yes | No | 6 |
| Wang (2021) | Yes | Yes | Yes | No | Yes | No | Yes | Yes | Unclear | Yes | Unclear | 7 |
| Li (2021) | Yes | Yes | Yes | No | Yes | No | Yes | Yes | Unclear | Yes | Unclear | 7 |
| Carrillo-Palau (2021) | Yes | Yes | No | Yes | Yes | No | No | Yes | Unclear | Yes | Unclear | 6 |
| Vrdolijak (2020) | Yes | Yes | Yes | No | Yes | Yes | Yes | Yes | Unclear | Yes | Unclear | 8 |
| Brinc (2020) | Yes | Yes | Yes | No | Yes | No | No | Yes | Unclear | Yes | Unclear | 6 |
| Dragasevic (2019) | Yes | Yes | No | No | Yes | No | No | Yes | Unclear | Yes | Unclear | 5 |
| Sahin (2019) | Yes | Yes | Yes | No | Yes | No | No | Yes | Unclear | Yes | Unclear | 6 |
| Qiao (2019) | Yes | Yes | Yes | Yes | Yes | No | No | Yes | Unclear | Yes | Unclear | 7 |
| Iwakawa (2019) | Yes | Yes | Yes | No | Yes | No | No | No | Unclear | Yes | Unclear | 5 |
| Ae Kang  (2019) | Yes | Yes | Yes | No | Yes | No | No | Yes | No | Yes | Yes | 7 |
| Grzybowska-Chlebowczyk (2018) | Yes | Yes | Yes | No | Yes | Yes | No | No | Unclear | Yes | Unclear | 6 |
| De Fatima Adorne (2016) | Yes | Yes | Yes | No | Yes | No | Yes | No | No | Yes | No | 6 |
| Wada (2015) | Yes | Yes | Yes | No | Yes | No | No | Yes | Unclear | Yes | Unclear | 6 |
| Liu (2013) | Yes | Yes | Yes | No | Yes | No | No | Yes | Unclear | Yes | Unclear | 6 |
| Akdogan (2013) | Yes | Yes | No | Yes | Yes | No | Yes | Yes | Unclear | Yes | Unclear | 7 |
| Yorulmaz (2011) | Yes | Yes | No | No | Yes | No | No | Yes | Unclear | Yes | Unclear | 5 |
| Kuwabara (2011) | Yes | Yes | No | No | Yes | No | No | Yes | Unclear | Yes | Unclear | 5 |
| Sappati Biyyani (2010) | Yes | Yes | Yes | No | Yes | No | Yes | Yes | Unclear | Yes | Unclear | 7 |
| Mijac (2010) | Yes | Yes | No | Yes | Yes | Yes | Yes | Yes | Unclear | Yes | Unclear | 8 |
| Romanato (2009) | Yes | Yes | Yes | No | Yes | No | No | Yes | Unclear | Yes | Unclear | 6 |
| Figler (2007) | Yes | Yes | No | No | Yes | No | Yes | Yes | Unclear | Yes | Unclear | 6 |
| Tajika (2004) | Yes | Yes | Yes | No | Yes | No | Yes | Yes | Unclear | Yes | Unclear | 7 |
| **Notes**: Q1: Define the source of information (survey, record review); Q2: List inclusion and exclusion criteria for exposed and unexposed subjects (cases and controls) or refer to previous publications; Q3: Indicate time period used for identifying patients; Q4: Indicate whether or not subjects were consecutive if not population-based; Q5: Indicate if evaluators of subjective components of study were masked to other aspects of the status of the participants; Q6: Describe any assessments undertaken for quality assurance purposes (e.g., test/retest of primary outcome measurements); Q7: Explain any patient exclusions from analysis; Q8: Describe how confounding was assessed and/or controlled; Q9: If applicable, explain how missing data were handled in the analysis; Q10: Summarize patient response rates and completeness of data collection; Q11: Clarify what follow-up, if any, was expected and the percentage of patients for which incomplete data or follow-up was obtained. | | | | | | | | | | | | |
