# Supplementary material for: Association of serum lipids with inflammatory bowel disease: a systematic review and meta-analysis
Source: Front Med (Lausanne). 2023 Aug 24;10:1198988. doi: 10.3389/fmed.2023.1198988 (PMC10484721; doi:10.3389/fmed.2023.1198988)
Supplement: Supplementary file 3 [file Table_3.docx]

| **Supplementary Table S3. Results of meta-regression analyses.** | |
| --- | --- |
| **Variables** | **P value** |
| **TC level** |  |
| *IBD vs. Healthy controls* | |
| Study design (Case-control versus cross-sectional versus cohort) | 0.401 |
| Publication year (After 2010 versus before 2010) | 0.163 |
| Region (Asia versus Europe versus America versus Oceania) | 0.517 |
| Sample size (>100 versus <=100) | ***0.041*** |
| Age and gender matched between two groups (Matched versus unmatched) | 0.753 |
| *CD vs. Healthy controls* | |
| Study design (Case-control versus cross-sectional versus cohort) | 0.531 |
| Publication year (After 2010 versus before 2010) | 0.458 |
| Region (Asia versus Europe versus America versus Oceania) | 0.396 |
| Sample size (>100 versus <=100) | 0.137 |
| Age and gender matched between two groups (Matched versus unmatched) | 0.199 |
| *UC vs. Healthy controls* | |
| Study design (Case-control versus cross-sectional versus cohort) | 0.912 |
| Publication year (After 2010 versus before 2010) | 0.535 |
| Region (Asia versus Europe versus America versus Oceania) | 0.064 |
| Sample size (>100 versus <=100) | ***0.039*** |
| Age and gender matched between two groups (Matched versus unmatched) | 0.912 |
| *CD vs. UC* | |
| Study design (Case-control versus cross-sectional versus cohort) | ***0.017*** |
| Publication year (After 2010 versus before 2010) | 0.701 |
| Region (Asia versus Europe versus America versus Oceania) | ***0.041*** |
| Sample size (>100 versus <=100) | 0.106 |
| **HDL level** | |
| *IBD vs. Healthy controls* | |
| Study design (Case-control versus cross-sectional versus cohort) | 0.267 |
| Publication year (After 2010 versus before 2010) | 0.990 |
| Region (Asia versus Europe versus America versus Oceania) | 0.197 |
| Sample size (>100 versus <=100) | 0.439 |
| Age and gender matched between two groups (Matched versus unmatched) | 0.902 |
| *CD vs. Healthy controls* | |
| Study design (Case-control versus cross-sectional versus cohort) | 0.982 |
| Publication year (After 2010 versus before 2010) | 0.062 |
| Region (Asia versus Europe versus America versus Oceania) | 0.563 |
| Sample size (>100 versus <=100) | 0.343 |
| Age and gender matched between two groups (Matched versus unmatched) | 0.582 |
| *UC vs. Healthy controls* | |
| Study design (Case-control versus cross-sectional versus cohort) | 0.901 |
| Publication year (After 2010 versus before 2010) | 0.362 |
| Region (Asia versus Europe versus America versus Oceania) | 0.120 |
| Sample size (>100 versus <=100) | 0.711 |
| Age and gender matched between two groups (Matched versus unmatched) | 0.516 |
| *CD vs. UC* | |
| Study design (Case-control versus cross-sectional versus cohort) | 0.290 |
| Publication year (After 2010 versus before 2010) | ***0.012*** |
| Region (Asia versus Europe versus America versus Oceania) | 0.174 |
| Sample size (>100 versus <=100) | 0.626 |
| **LDL level** | |
| *IBD vs. Healthy controls* | |
| Study design (Case-control versus cross-sectional versus cohort) | 0.858 |
| Publication year (After 2010 versus before 2010) | 0.184 |
| Region (Asia versus Europe versus America versus Oceania) | 0.900 |
| Sample size (>100 versus <=100) | 0.178 |
| Age and gender matched between two groups (Matched versus unmatched) | 0.421 |
| *CD vs. Healthy controls* | |
| Study design (Case-control versus cross-sectional versus cohort) | 0.568 |
| Publication year (After 2010 versus before 2010) | 0.120 |
| Region (Asia versus Europe versus America versus Oceania) | 0.989 |
| Sample size (>100 versus <=100) | 0.132 |
| Age and gender matched between two groups (Matched versus unmatched) | 0.647 |
| *UC vs. Healthy controls* | |
| Study design (Case-control versus cross-sectional versus cohort) | 0.483 |
| Publication year (After 2010 versus before 2010) | 0.159 |
| Region (Asia versus Europe versus America versus Oceania) | 0.211 |
| Sample size (>100 versus <=100) | 0.271 |
| Age and gender matched between two groups (Matched versus unmatched) | 0.957 |
| *CD vs. UC* | |
| Study design (Case-control versus cross-sectional versus cohort) | 0.934 |
| Publication year (After 2010 versus before 2010) | 0.225 |
| Region (Asia versus Europe versus America versus Oceania) | 0.934 |
| Sample size (>100 versus <=100) | 0.786 |
| **TG level** | |
| *IBD vs. Healthy controls* | |
| Study design (Case-control versus cross-sectional versus cohort) | 0.615 |
| Publication year (After 2010 versus before 2010) | 0.263 |
| Region (Asia versus Europe versus America versus Oceania) | 0.122 |
| Sample size (>100 versus <=100) | 0.520 |
| Age and gender matched between two groups (Matched versus unmatched) | 0.483 |
| *CD vs. Healthy controls* | |
| Study design (Case-control versus cross-sectional versus cohort) | 0.782 |
| Publication year (After 2010 versus before 2010) | 0.848 |
| Region (Asia versus Europe versus America versus Oceania) | 0.273 |
| Sample size (>100 versus <=100) | 0.531 |
| Age and gender matched between two groups (Matched versus unmatched) | 0.704 |
| *UC vs. Healthy controls* | |
| Study design (Case-control versus cross-sectional versus cohort) | 0.794 |
| Publication year (After 2010 versus before 2010) | 0.594 |
| Region (Asia versus Europe versus America versus Oceania) | 0.602 |
| Sample size (>100 versus <=100) | 0.884 |
| Age and gender matched between two groups (Matched versus unmatched) | 0.977 |
| *CD vs. UC* | |
| Study design (Case-control versus cross-sectional versus cohort) | 0.228 |
| Publication year (After 2010 versus before 2010) | 0.119 |
| Region (Asia versus Europe versus America versus Oceania) | 0.517 |
| Sample size (>100 versus <=100) | 0.174 |
| **Abbreviations:** IBD, inflammatory bowel disease; UC, ulcerative colitis; CD, Crohn’s disease; TC, total cholesterol; HDL-c, high density lipoprotein cholesterol; LDL-c, low density lipoprotein cholesterol; TG, triglyceride. | |
