# Supplementary material for: Association of serum lipids with inflammatory bowel disease: a systematic review and meta-analysis
Source: Front Med (Lausanne). 2023 Aug 24;10:1198988. doi: 10.3389/fmed.2023.1198988 (PMC10484721; doi:10.3389/fmed.2023.1198988)
Supplement: Supplementary file 4 [file Table_4.docx]

| **Supplementary Table S4. Publication bias.** | | | | | |
| --- | --- | --- | --- | --- | --- |
| **Endpoints** | **Number of included studies** | **t** | **P** | **95%Confidence interval** | |
| *IBD versus Healthy controls* | | | | | |
| TC level | 36 | -1.80 | 0.081 | -3.700 | 0.226 |
| HDL-c level | 29 | 0.71 | 0.484 | -1.890 | 3.888 |
| LDL-c level | 29 | 0.30 | 0.765 | -2.333 | 3.138 |
| TG level | 33 | 1.96 | 0.058 | -0.726 | 3.895 |
| *CD versus Healthy controls* | | | | | |
| TC level | 17 | 1.89 | 0.078 | -3.788 | 6.408 |
| HDL-c level | 12 | 1.72 | 0.117 | -0.918 | 7.068 |
| LDL-c level | 11 | 5.17 | ***0.001*** | 3.228 | 8.256 |
| TG level | 15 | 1.65 | 0.123 | -1.126 | 8.370 |
| *UC versus Healthy controls* | | | | | |
| TC level | 14 | 0.34 | 0.741 | -3.299 | 4.514 |
| HDL-c level | 12 | -1.32 | 0.215 | -3.932 | 1.003 |
| LDL-c level | 11 | 0.08 | 0.939 | -4.228 | 4.535 |
| TG level | 13 | 1.95 | 0.077 | -0.440 | 7.268 |
| *CD versus UC* | | | | | |
| TC level | 21 | 0.52 | 0.611 | -2.082 | 3.448 |
| HDL-c level | 15 | 0.70 | 0.498 | -1.125 | 2.199 |
| LDL-c level | 13 | 1.42 | 0.183 | -1.366 | 6.352 |
| TG level | 18 | 1.61 | 0.126 | -0.439 | 3.248 |
| **Abbreviations:** IBD, inflammatory bowel disease; UC, ulcerative colitis; CD, Crohn’s disease; TC, total cholesterol; HDL-c, high density lipoprotein cholesterol; LDL-c, low density lipoprotein cholesterol; TG, triglyceride. | | | | | |
