# Supplementary material for: Association of serum lipids with inflammatory bowel disease: a systematic review and meta-analysis
Source: Front Med (Lausanne). 2023 Aug 24;10:1198988. doi: 10.3389/fmed.2023.1198988 (PMC10484721; doi:10.3389/fmed.2023.1198988)

**Supplementary Material 2:**

Search criteria

| Database | Search term |
| --- | --- |
| PubMed: 637 hits | (("cholesterol"[MeSH]) OR ("cholesterol" OR "cholesterol s" OR "cholesterol" OR "cholesterols") OR ("Triglycerides"[MeSH]) OR ("Triacylglycerols" OR "Triacylglycerol" OR "Triglyceride") OR ("Cholesterol, LDL" [MeSH]) OR ("Low Density Lipoprotein Cholesterol" OR "beta-Lipoprotein Cholesterol" OR "Cholesterol, beta-Lipoprotein" OR "beta Lipoprotein Cholesterol" OR "LDL Cholesterol" OR "Cholesteryl Linoleate, LDL" OR "LDL Cholesteryl Linoleate") OR ("Cholesterol, HDL"[MeSH]) OR ("alpha-Lipoprotein Cholesterol" OR "Cholesterol, alpha-Lipoprotein" OR "alpha Lipoprotein Cholesterol" OR "HDL Cholesterol" OR "High Density Lipoprotein Cholesterol" OR "Cholesterol, HDL2" OR "HDL2 Cholesterol" OR "HDL(2) Cholesterol" OR "Cholesterol, HDL3" OR "HDL3 Cholesterol" OR "HDL(3) Cholesterol")) AND (("Colitis, Ulcerative"[MeSH]) OR ("colitis ulcerative" OR "ulcerative colitis" OR "Colitis Gravis") OR ("Crohn Disease"[Mesh]) OR ("crohn" OR "crohn s" OR "crohns") OR ("Inflammatory Bowel Diseases"[Mesh]) OR ("Inflammatory Bowel Disease" OR "Bowel Diseases, Inflammatory")) |
| Embase: 2416 hits | #1 'cholesterol'/exp OR 'cholesterol s' OR 'cholesterol' OR 'cholesterols' OR 'triglycerides'/exp OR 'triacylglycerols' OR 'triacylglycerol' OR 'triglyceride' OR 'cholesterol, ldl'/exp OR 'low density lipoprotein cholesterol' OR 'beta-lipoprotein cholesterol' OR 'cholesterol, beta-lipoprotein' OR 'beta lipoprotein cholesterol' OR 'ldl cholesterol' OR 'cholesteryl linoleate, ldl' OR 'ldl cholesteryl linoleate' OR 'cholesterol, hdl'/exp OR 'alpha-lipoprotein cholesterol' OR 'cholesterol, alpha-lipoprotein' OR 'alpha lipoprotein cholesterol' OR 'hdl cholesterol' OR 'high density lipoprotein cholesterol' OR 'cholesterol, hdl2' OR 'hdl2 cholesterol' OR 'hdl(2) cholesterol' OR 'cholesterol, hdl3' OR 'hdl3 cholesterol' OR 'hdl(3) cholesterol'  #2 'colitis, ulcerative'/exp OR 'colitis ulcerative' OR 'ulcerative colitis' OR 'colitis gravis' OR 'crohn disease'/exp OR 'crohn' OR 'crohn s' OR 'crohns' OR 'inflammatory bowel diseases'/exp OR 'inflammatory bowel disease' OR 'bowel diseases, inflammatory'  #3 #1 AND #2 |
| Cochrane Library:  182 hits | #1 MeSH descriptor: [Triglycerides] explode all trees  #2 triglycerid OR triglycerides OR triglyceride OR triglycerids  #3 #1 OR #2  #4 MeSH descriptor: [Cholesterol, LDL] explode all trees  #5 Low Density Lipoprotein Cholesterol OR beta-Lipoprotein Cholesterol OR Cholesterol, beta-Lipoprotein OR beta Lipoprotein Cholesterol OR LDL Cholesterol OR Cholesteryl Linoleate, LDL OR LDL Cholesteryl Linoleate  #6 #4 OR #5  #7 MeSH descriptor: [Cholesterol, HDL] explode all trees  #8 alpha-Lipoprotein Cholesterol OR Cholesterol, alpha-Lipoprotein OR alpha Lipoprotein Cholesterol OR HDL Cholesterol OR High Density Lipoprotein Cholesterol OR Cholesterol, HDL2 OR HDL2 Cholesterol OR HDL(2) Cholesterol OR Cholesterol, HDL3 OR HDL3 Cholesterol OR HDL(3) Cholesterol  #9 #7 OR #8  #10 MeSH descriptor: [Cholesterol] explode all trees  #11 cholesterol OR cholesterol s OR cholesterole OR cholesterols  #12 #10 OR #11  #13 #3 OR #6 OR #9 OR #12  #14 MeSH descriptor: [Colitis, Ulcerative] explode all trees  #15 colitis ulcerative OR ulcerative colitis OR Colitis Gravis  #16 #14 OR #15  #17 MeSH descriptor: [Crohn Disease] explode all trees  #18 crohn OR crohn s OR crohns  #19 #17 OR #18  #20 MeSH descriptor: [Inflammatory Bowel Diseases] explode all trees  #21 Inflammatory Bowel Disease OR Bowel Diseases, Inflammatory  #22 #20 OR #21  #23 #16 OR #19 OR #22  #24 #13 AND #23 |


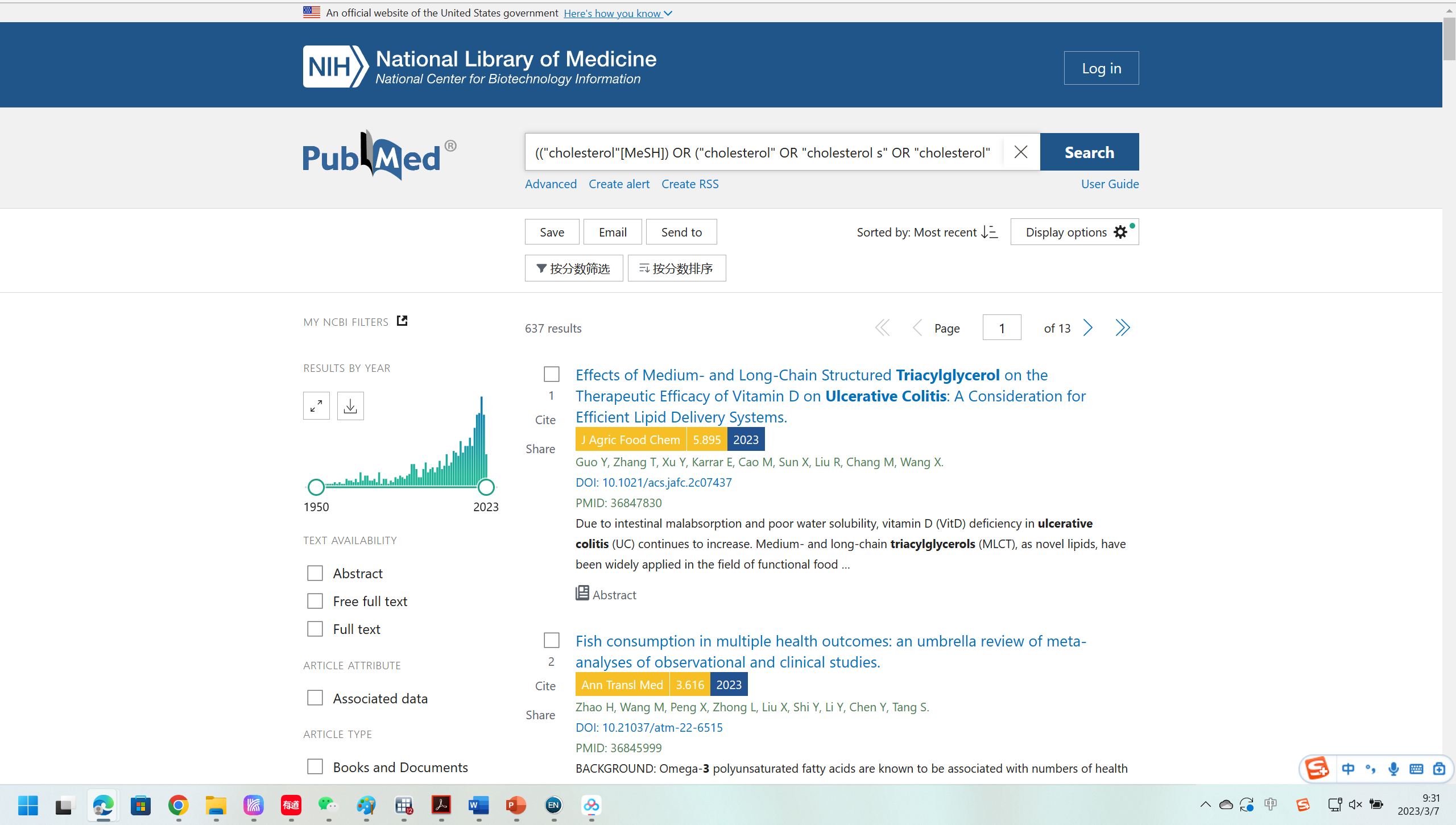


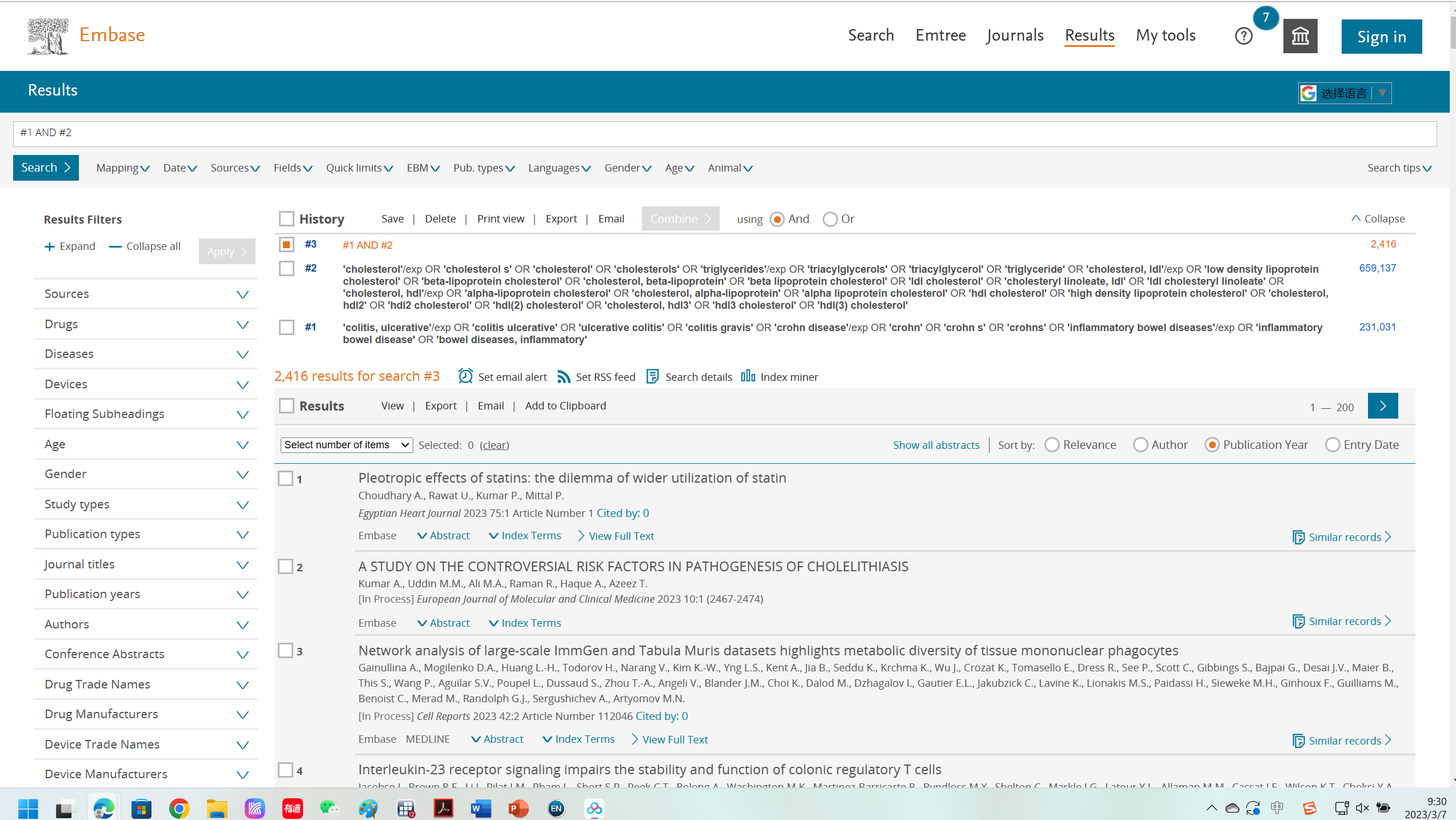


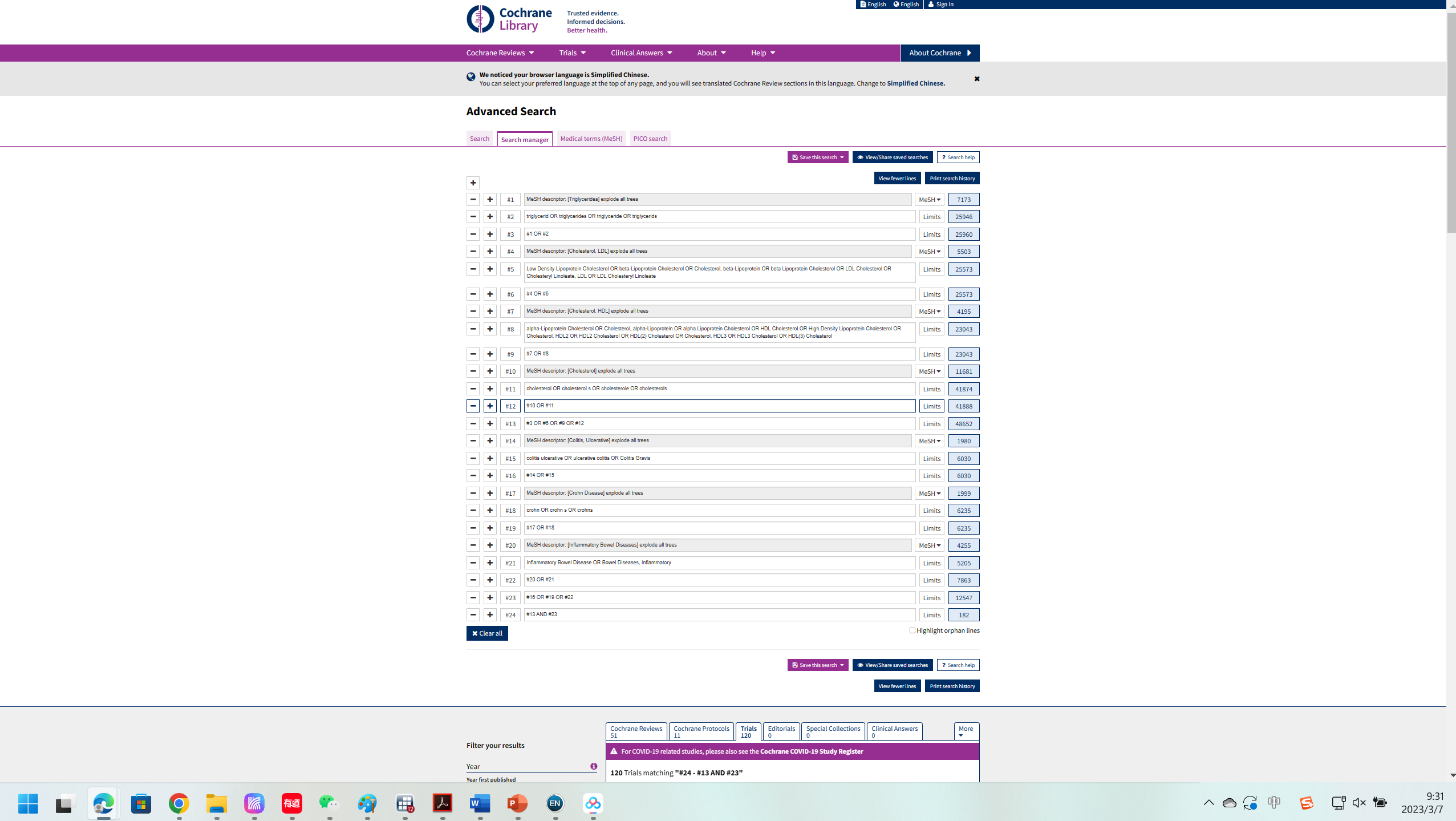

Supplement: Supplementary file 6 [file Data_Sheet_2.docx]
