# Supplementary figures and images for: Association of serum lipids with inflammatory bowel disease: a systematic review and meta-analysis
Source: Front Med (Lausanne). 2023 Aug 24;10:1198988. doi: 10.3389/fmed.2023.1198988 (PMC10484721; doi:10.3389/fmed.2023.1198988)

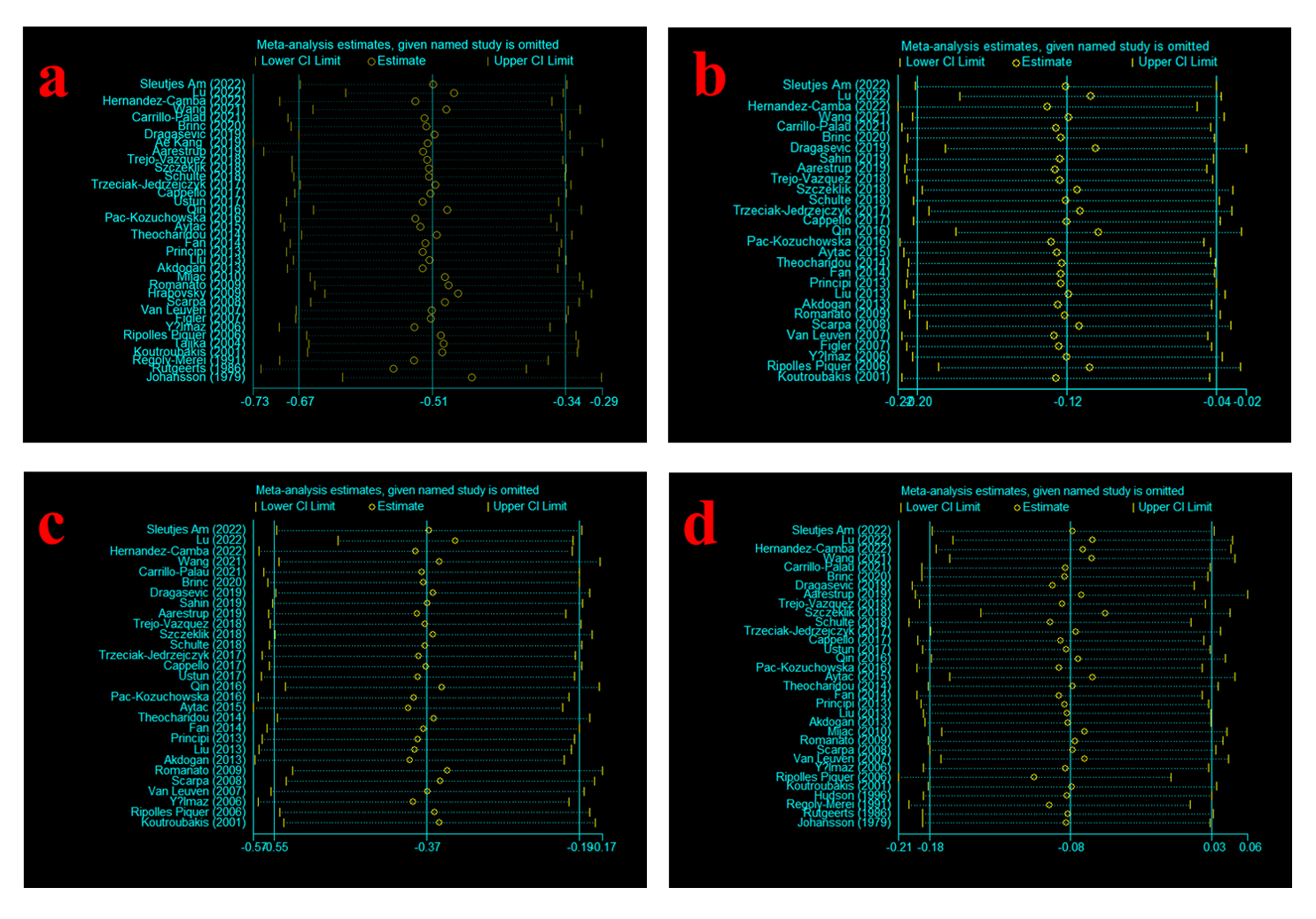

Supplement: Supplementary file 7 [file Image_1.JPEG]

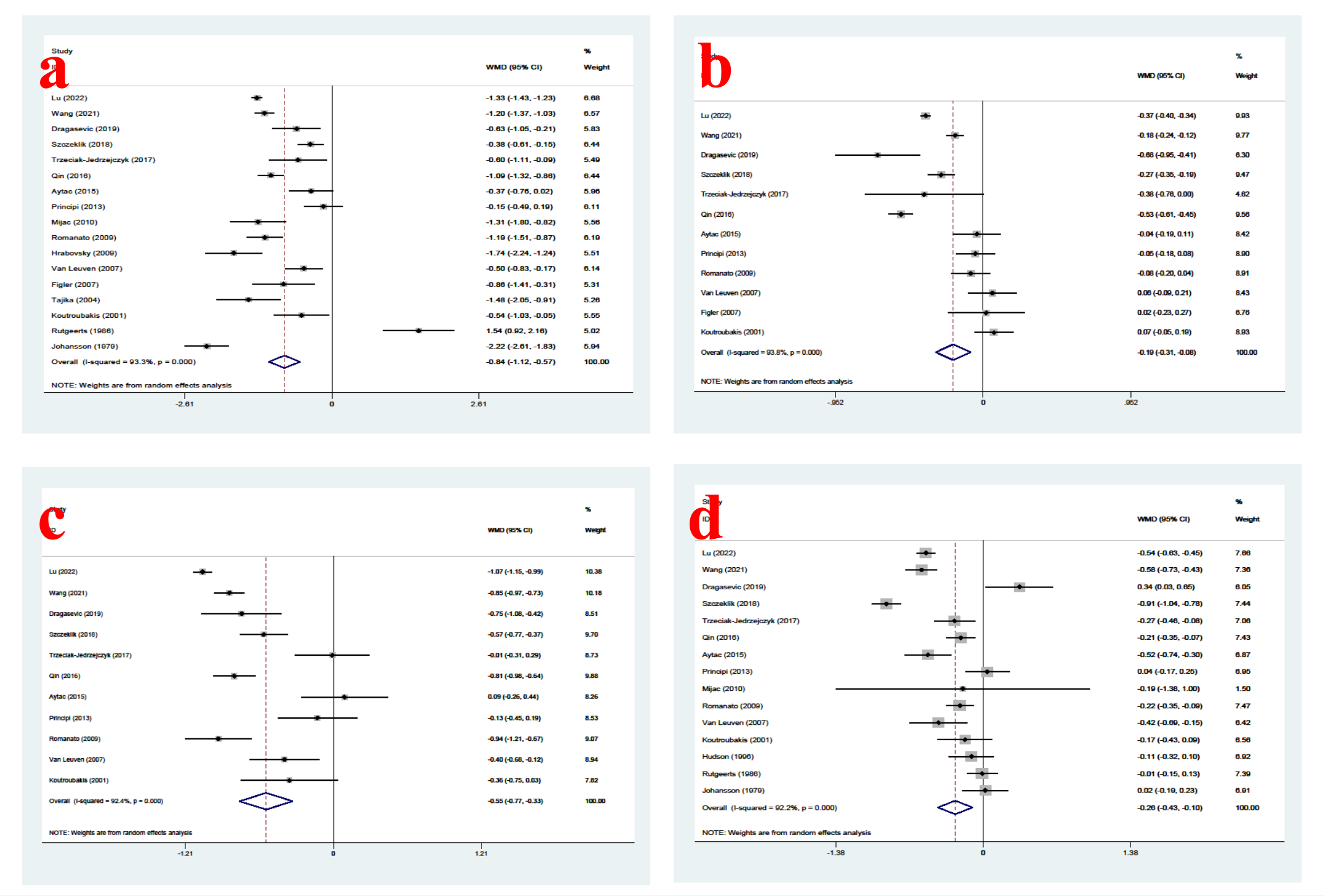

Supplement: Supplementary file 8 [file Image_2.JPEG]

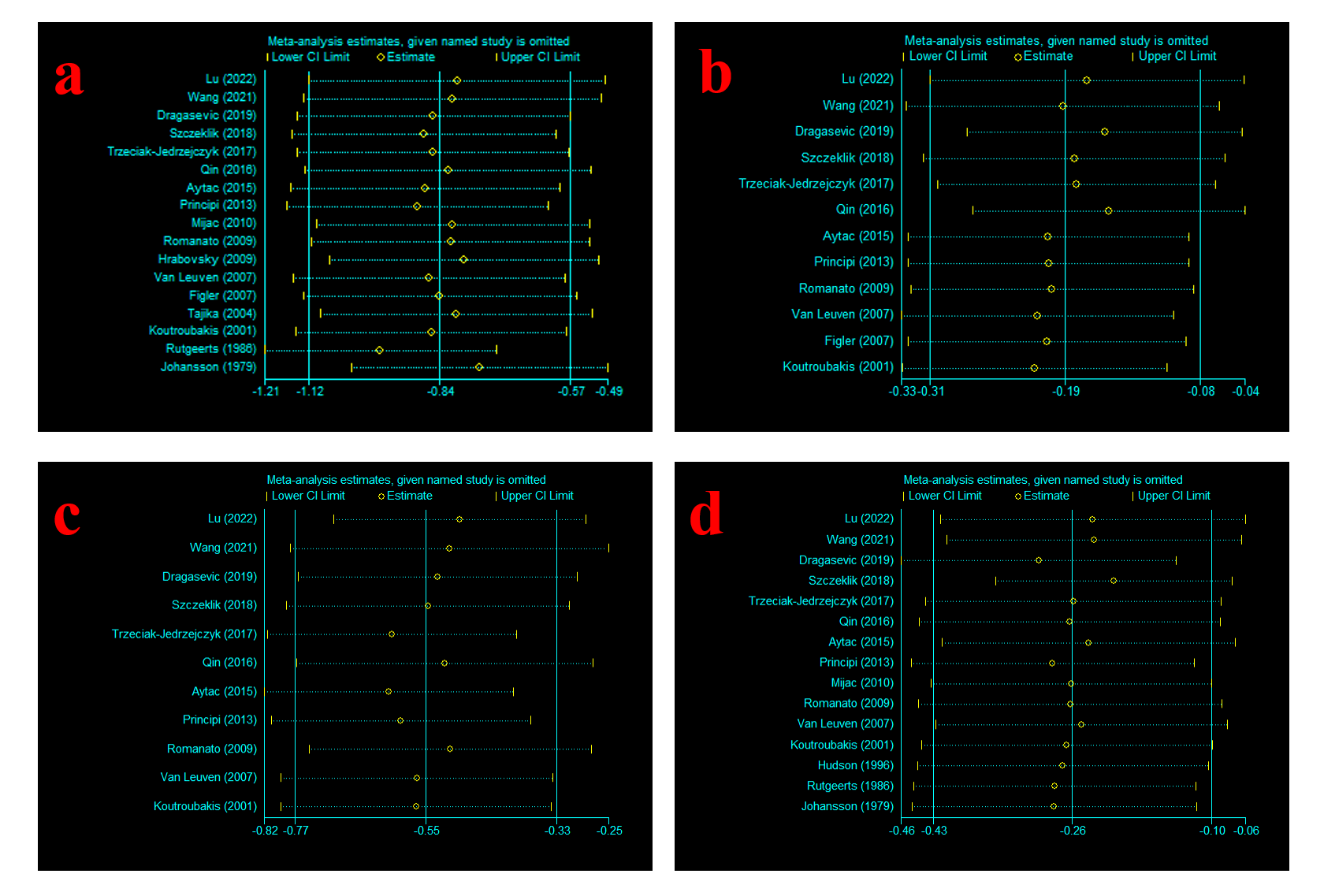

Supplement: Supplementary file 9 [file Image_3.JPEG]

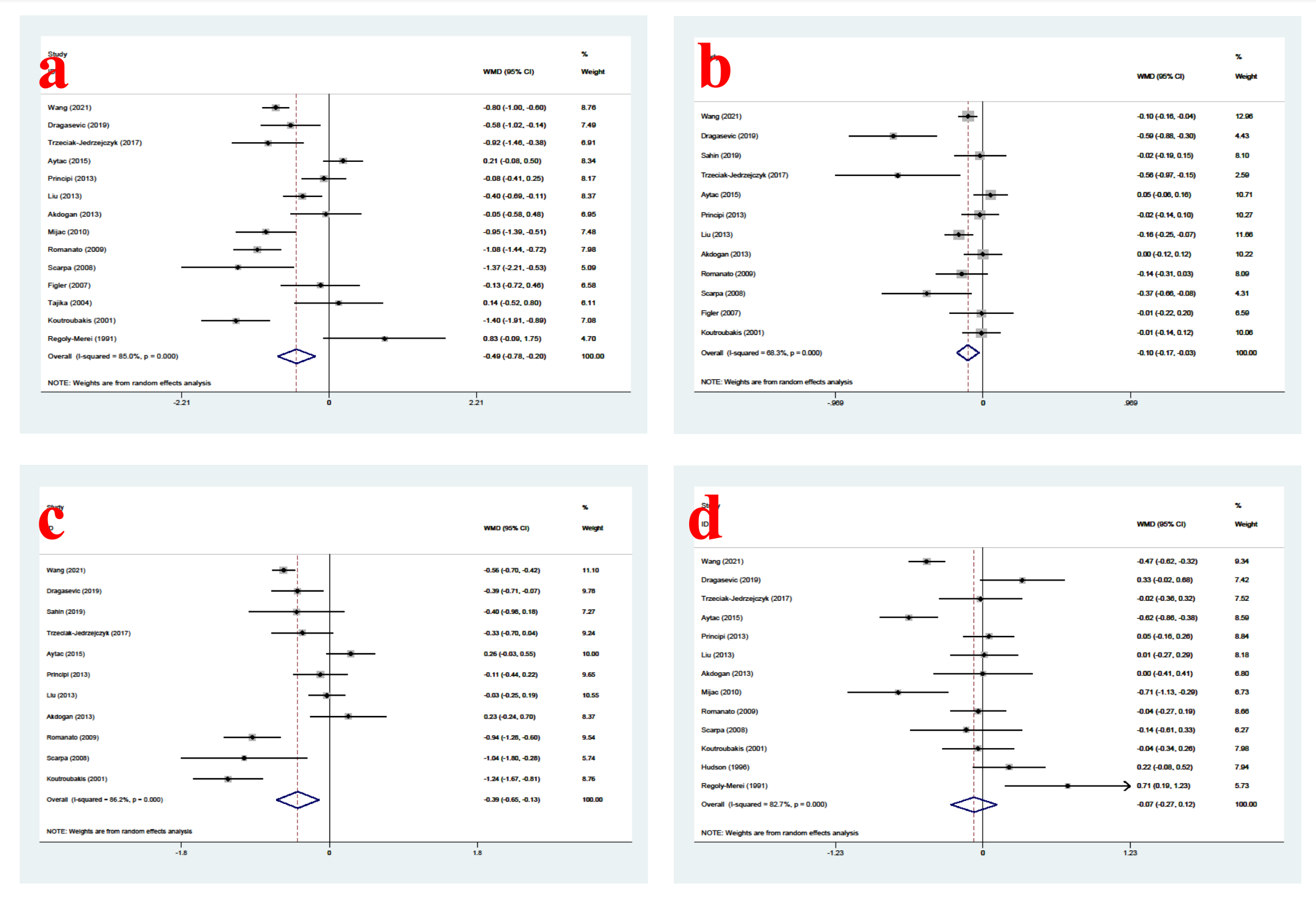

Supplement: Supplementary file 10 [file Image_4.JPEG]

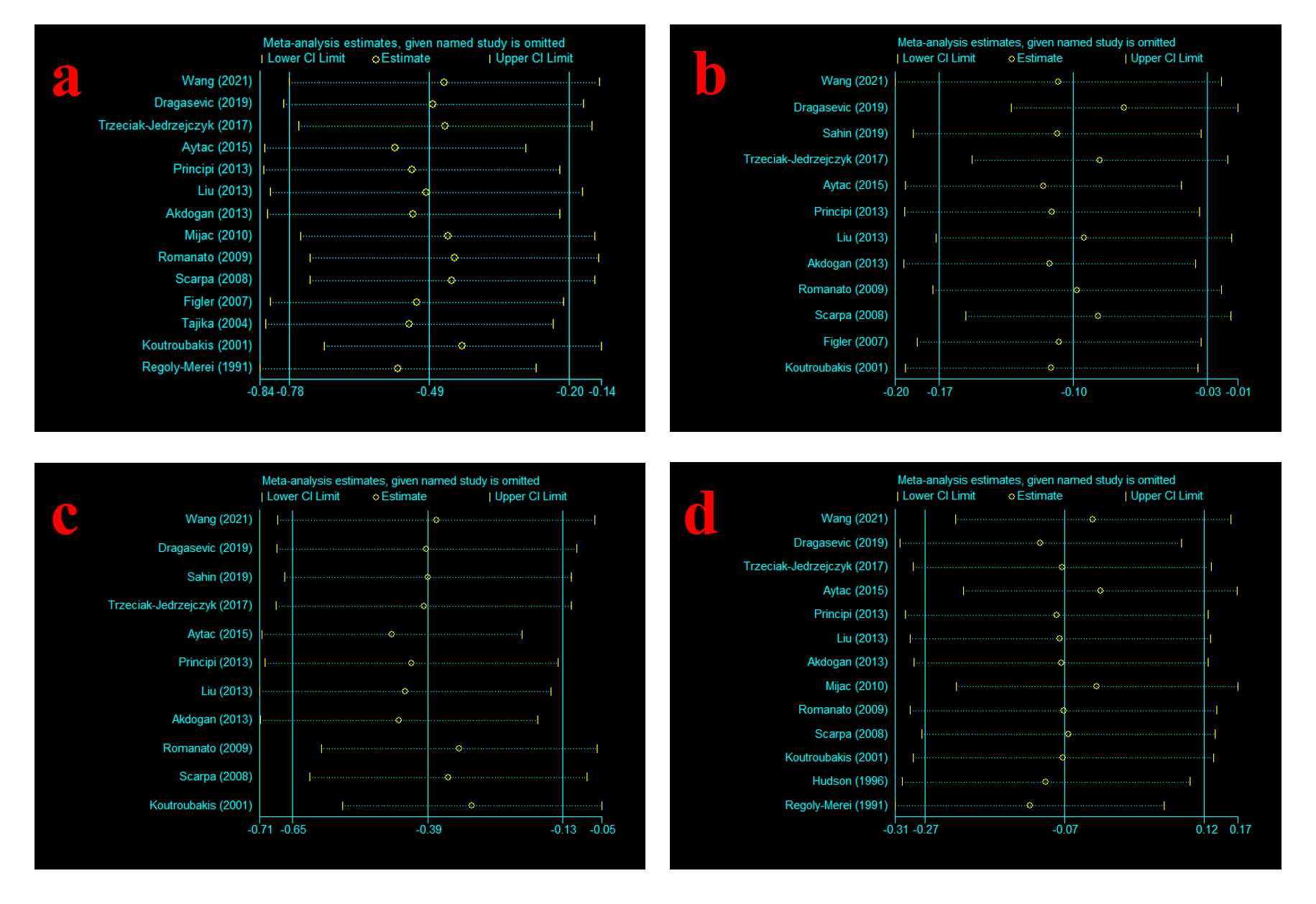

Supplement: Supplementary file 11 [file Image_5.JPEG]

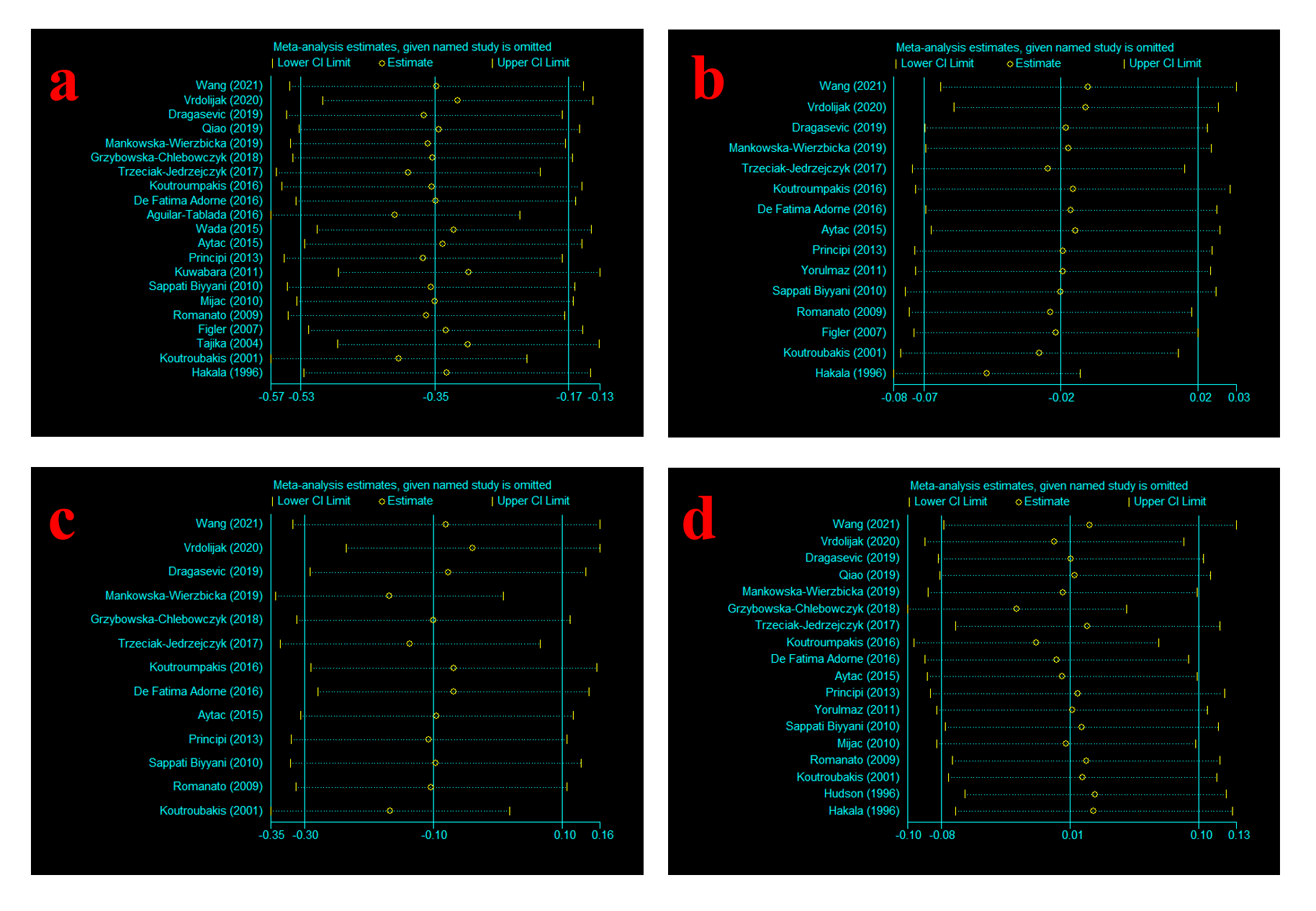

Supplement: Supplementary file 12 [file Image_6.JPEG]
